# Introduction to Traditional Medicine and Their Role in Prevention and Treatment of Emerging and Re-Emerging Diseases

**DOI:** 10.3390/biom12101442

**Published:** 2022-10-09

**Authors:** Syed A. A. Rizvi, George P. Einstein, Orien L. Tulp, Frantz Sainvil, Rolando Branly

**Affiliations:** 1 College of Biomedical Sciences, Larkin University, Miami, FL 33169, USA; 2 College of Medicine, University of Science, Arts and Technology, Olveston P.O. Box 506, UK; 3 Physical Sciences Department, Broward College, Davie, FL 33332, USA

**Keywords:** natural products, infectious disease, COVID-19, pandemic, immunity

## Abstract

Infectious diseases have been a threat to human health globally. The relentless efforts and research have enabled us to overcome most of the diseases through the use of antiviral and antibiotic agents discovered and employed. Unfortunately, the microorganisms have the capability to adapt and mutate over time and antibiotic and antiviral resistance ensues. There are many challenges in treating infections such as failure of the microorganisms to respond to the therapeutic agents, which has led to more chronic infections, complications, and preventable loss of life. Thus, a multidisciplinary approach and collaboration is warranted to create more potent, effective, and versatile therapies to prevent and eradicate the old and newly emerging diseases. In the recent past, natural medicine has proven its effectiveness against various illnesses. Most of the pharmaceutical agents currently used can trace their origin to the natural products in one way, shape, or form. The full potential of natural products is yet to be realized, as numerous natural resources have not been explored and analyzed. This merits continuous support in research and analysis of ancient treatment systems to explore their full potential and employ them as an alternative or principal therapy.

## 1. Introduction

It has been over two years since the COVID-19 pandemic took the world by a storm, killing millions of people, destroying economies, and causing misery. Given that almost all nations suffered in some form and scientists worldwide came together, a comprehensive cure is yet to be found. However, many antivirals and vaccines are being used currently with varying degrees of success [1]. Prevention of the disease is the only measure to avoid its ferocious effects. The immune system can be modulated using multiple vaccines that have been developed, and also with natural supplements. During the COVID-19 pandemic, communities around the world have also been advised to adhere to the set health protocols in their localities to avoid getting infected and transmitting the virus to others. Nutrition-based interventions have shown to play a pivotal role in preventing and managing viral infections, particularly those affecting the respiratory system [2].

As more research is carried out on the effects of the nutrition-based interventions in the treatment and management of COVID-19, publications that discuss the role of traditional medicine, botanicals, and nutraceuticals are plentiful [3]. Studies have suggested that the suppression of immunity is discernible in individuals who have micronutrient deficiencies [4]. The immune system functions are greatly influenced by the T cell-mediated immune responses and the adaptive responses of the antibodies. Enhanced immunity enables the host to fight viral respiratory infections adequately [2].

Individuals around the world were overwhelmed with the information overload while the search for a vaccine and a specific antiviral was still underway, and not much was known about the novel virus itself. Unfortunately, much of the information available on the internet was not necessarily data-driven or research-based. Statistics showed that in March of 2020, when the surge of the COVID-19 infections was at the zenith, the purchase of elderberry and zinc supplements was on the rise too; this had increased by 255% and 415%, respectively [5]. The governments and other pertinent health organizations of a country had the role of verifying the information that their citizens were exposed to. Multiple organizations and companies had been warned on issuing misleading and false information related to the cure and the treatment of COVID-19 [6].

**Figure 1:**
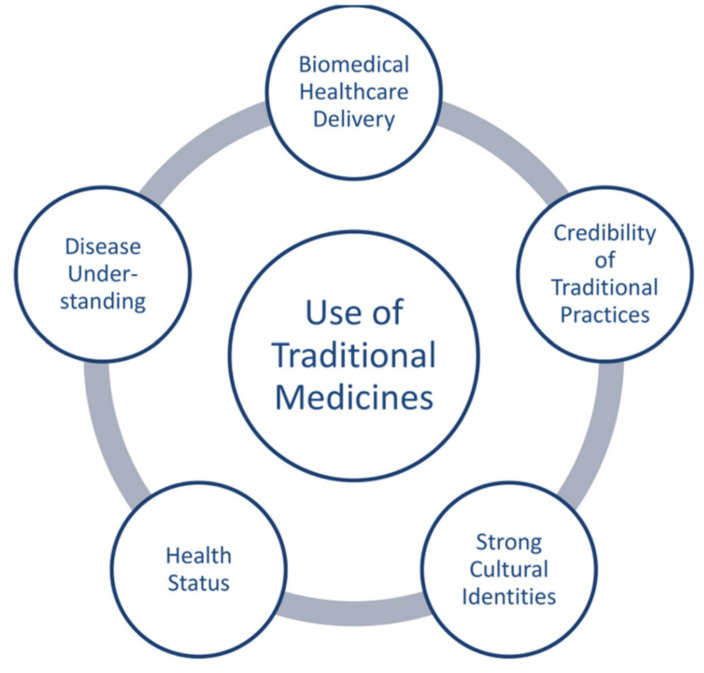
“Determinants for the use of Traditional Medicines”. Reprinted under the terms of the Creative Commons Attribution License from, Stanifer, J.W.; Patel, U.D.; Karia, F.; et al. The determinants of traditional medicine use in Northern Tanzania: a mixed-methods study. *PLoS ONE* **2015**, *10*(4), e0122638. doi:10.1371/journal.pone.0122638.

Using natural products, which is part of various traditional medical systems to prevent and/or treat diseases, dates back thousands of years in different parts of the world [7]. The popularity of natural therapy has been on the rise all around for maintaining good health, as it plays a role in the prevention of diseases and illnesses. It has been used as both complementary and conventional therapy in managing both curable and incurable diseases [7]. More emphasis has been placed in using natural products as complementary and alternative therapies (Figure 1). Approximately 25% of the contemporary drugs are derived from plants and 60% of the anti-infectious drugs in the market and under development are of natural origin [8]. About 75% of liver disease patients in the US and Europe consume herbal medications. In Germany alone, the use of silymarin, a hepatoprotective and antioxidant natural product, has surpassed $180 million [9].

Some natural products are abundantly available without synthesizing them in the laboratories; this has reduced the production costs [10]. Multiple phytochemicals and their derivatives have been studied and have exhibited the potential in becoming successful remedies. Some examples are glycyrrhizin, which has been used in the management of viral hepatitis; ellagic acid, which has been used to manage fibrosis; and hepatitis B has been managed with phyllanthin [11]. The compounds have different mechanisms of action and characteristics, usually presenting an intrinsic beneficial or toxic effect, and have been used as hepatoprotective agents and as antioxidants [12].

Studies show that collectively, plants produce more than 100,000 secondary metabolites that can be differentiated based on their composition, biosynthetic pathway, or chemical structure [13]. Three groups have been used as a simple classification of these compounds. The first group is the terpenoids synthesized from mevalonic acid and composed of only carbon and hydrogen. The second group is the phenolic compounds synthesized from simple sugars composed of benzene rings, oxygen, and hydrogen. Lastly, the alkaloids are nitrogen-containing compounds [14].

Not all potential benefits from natural products have been realized, as a vast majority of them have not been explored [15]. Based on the available studies and data, the continuous evaluation of various natural products will enhance our knowledge, perception, and attitude towards the use of plant-derived products as therapies for different conditions [15]. Herein, we explore traditional Chinese and Indian medicine, and other natural products, for preventing and managing microbial infections, including COVID-19.

## 2. Traditional Indian Medicine

### 2.1. Ayurveda

**Figure 2:**
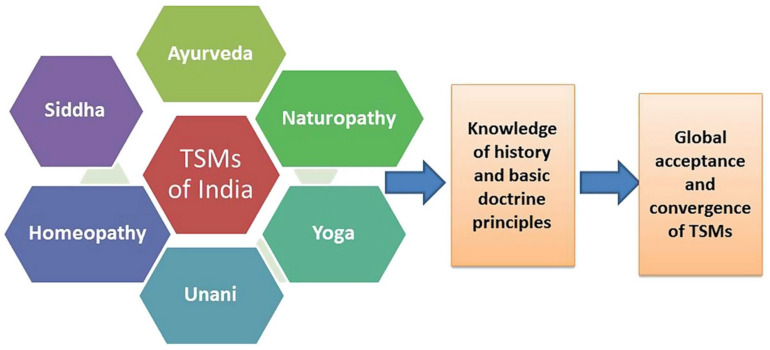
Traditional systems of medicine (TSMs) of India. Reproduced with permission from Elsevier. Figure obtained from an article published in Journal of Traditional and Complementary Medicine, 7 (1), Jaiswal YS, Williams LL, A glimpse of Ayurveda—The forgotten history and principles of Indian traditional medicine, 50–53, Copyright Elsevier (2016).

Traditional Indian medicine has been in existence since antiquity and is inarguably among the most ancient systems of traditional medicine still in existence [16]. It has been proven to date back tens of thousands of years; the longest period determined for any traditional system (Figure 2).

Some other arguments have held that the Ayurveda system is the core base of the widely renowned Tibetan medicine, traditional Chinese medicine, and the Unani (Arabic) medicine systems [17]. All these systems ascribe to a common and accepted body–mind–spirit orientation; that holds that disease and health are a product of the interaction of these three fragments. They are also based on the heating and cooling energy processes. There is also an apparent relationship between yoga and Ayurveda [18]. The popularity of both has been on the rise in current times; they are almost inseparable; one cannot be mentioned without the other. History has shown that the spiritual teachers who first introduced Ayurveda combined it with other morally accepted practices such as vegetarianism, which has become acceptable as a harmonious pivotal system for the former, rather than an established holistic healing system which it truly is [19].

Since the introduction of Ayurveda in the West, the system has gained popularity and has been accepted as one of the prominent medical systems. Clinical trials, teachings, and writings related to Ayurveda have played a tremendous role in popularizing it. Some of the most prominent Westerners who have helped make the system known worldwide include David Frawley and Robert Svoboda, whose works have had an immense effect on the knowledge of the system. The relevance of Ayurveda in the modern world remains as it was since the commencement of printing of the protagonist classic texts that were compiled in the fifth and sixth centuries [20]. The recommendations and prescriptions are dynamic and not limited to specific people based on ethnicity, belief, or religion.

The influence of Ayurveda in the medical world has been so immense, it has come to be referred as ‘the mother of healing’. It goes without saying that for one to understand the basis of contemporary medicine, one needs first to understand the roots of Ayurveda [21]. Most of the natural products utilized in traditional Chinese medicine (TCM) and the modern-day era are also found in Ayurveda. However, even though traditional Indian medicine has been in existence for thousands of years, there is still more to be discovered with the application of modern technology and science. The world we live in is constantly evolving and so is our body, thus, to accommodate these changes. The systems of medicine available, including Ayurveda, will also change to meet the needs and gaps in these changes.

Regardless of the enthusiasm to find new and better remedies for various diseases and the vast armamentarium available to the medical community nowadays, challenges still exist, as there is a lack of sources of literature about the history and the basic principles of the systems in the different ethnicities [22]. This knowledge gap would have perhaps addressed issues that remain a mystery to this date.

The Ayurveda system holds that the whole universe consists of five main elements, being Vayu (air), Jala (Water), Aakash (space or ether), Prithvi (earth), and Teja (fire). The latter (referred to as Pancha Mahabhoota in Ayurveda) is composed of three vital body humors in different amounts. The three humors are collectively referred to as “Tridoshas,” including Vata dosha, Pitta dosha, and Kapha dosha. Each of the elements contain five sub-doshas that control the most important physiological functions of the body. According to this system, the human body contains Saptadhatus (seven tissues), Rakta (blood), Rasa (tissue fluids), Meda (fat and connective tissue), Asthi (bones), Mamsa (muscle), Majja (marrow), Shukra (semen) and three Malas (waste products) of the body, viz. Purusha (feces), Mutra (urine), and Sweda (sweat) [23]. The Vata dosha oversees the body’s electrolyte balance, transport of cellular components, and getting rid of waste products. Its effects on the body are resonated by dryness [24]. The different tissues interact with one another to maintain the normal physiological functioning of the body. The blood circulation and production of the important blood components in the body are performed by the Rakta Dhatu. On the other hand, the Mamsa Dhatu (Muscle tissue) maintains the support of the skeletal muscles for the adipose tissue (Meda Dhatu). The bones are made up by the Asthi Dhatu, while the Majja Dhatu consists of the fluids that oleate the bones and the bone marrow. The Shukra Dhatu controls the reproductive functions of the body [24].

The Pitta dosha regulates the body temperature, thirst, hunger, and coordination of the optic nerves. The Kapha dosha is affected by fatty and sweet foods; it enables the lubrication of the body’s joints for proper coordination. It is believed that the Vata controls the body’s catabolism; the Kapha oversees the metabolism, while the Kapha controls the anabolism of the body. For the body to maintain a healthy state, the three doshas and other related factors and their interactions must have a balance. If there are any significant imbalances between the three, disease or illness will likely result. A balance of the three doshas can be maintained if one follows the basic principles of divine wisdom; this is a major belief of the Ayurveda system [25].

The rich medicine systems in India comprises six systems, albeit Ayurveda is the most prominent, most widely accepted, practiced, and established. The other five systems of medicine include Siddha, Unani, Homeopathy, Yoga, and Naturopathy [26].

### 2.2. Siddha System

Protagonists and inheritors of the Siddha system of traditional medicine proclaim that it provides ‘Holistic health’ to all its users. The system provides rejuvenating, rehabilitative, promotive, and preventive care through a holistic and scientific approach. Siddha is rooted in “Citti,” which means achieving accomplishment, internal bliss, and perfection. The system entails philosophical concepts with four components: medical practice, yogic practice, iatrochemistry, and wisdom. These revolve around the intellectual, psychological, physical, and physiological aspects of all human beings. Some of them are regarded as the most fundamental unit of all the components of the body and the entire cosmos [27].

The Siddha system include theories of five elements (Aimpootham) that constitute the universe (in terms of properties), three forces/faults (Mukkuttram) that help maintaining homeostasis, and eight methods of examination (Envakai Thervukal) that help in proper diagnosis and treatment. The body’s physical components identified by this system are also highlighted in the Ayurveda system. The pathological and physiological aspects of these components have been described in the literature of this system and heavily relied upon in practice [28]. The cornerstone of the treatment of Siddha is based on a holistic approach. The system’s interventions aim to restore the normalcy of the vital factors of life. For instance, the taste of foods and the medicines used are considered important in treatment. The taste is accorded much attention, as it is the basis of selecting the drugs used for treatment. It has been used to treat both acute and chronic illnesses that include, but are not limited to, diabetes mellitus, obesity, hemiplegia parkinsonism, muscular disorders, skin diseases, digestive disorders, respiratory diseases, and arthritis [28].

### 2.3. Unani System

Unani is also known as Unani Tibb and is believed to have originated from Greece; its doctrines have been found in the writings of the ancient Greek physicians Galen and Hippocrates. The system was refined and developed with time through experimentation by the Arabs and was made prominent by the Muslim teacher Avicenna. Part of the medicine’s principles was adopted from this system during the Muslim political-religious regime in 632 CE. This system has been established through contributions from the Middle East and South Asia; it has become acceptable in different parts of the globe. Healing from the system relies upon principles of balance and harmony that include uniting the spiritual, mental, and physical realms together [29].

The system holds that the health of the body is maintained by the orderly arrangement of the seven vital physiological principles as stated by the Unani doctrine. These principles are arkan (elements), mizaj (temperament), akhlat (bodily humors), aaza (organs and systems), arwah (vital spirit), afaal (functions), and quwa (faculties or powers). All these components interact with one another to maintain the balance of the normal and natural makeup of the human body. Every human constitution has the capacity and power to regulate itself and maintain the seven components’ equilibrium [30].

There are four entities, thenar (fire), arz (earth), Hawa (air), and maa (water), that make up the basic components of the body and the entire creations of the Earth. The four entities can interact to create consequences that are predictable by man. The elements act upon and rely on each other; they continuously change deterioration and generation due to the body’s reaction to the medicines administered. Only skilled healers can observe such changes in the body [31].

The temperament states are the following four; moist, cold, dry, and hot. The four components are contained in balance by all the entities of the cosmos, such as plants, animals, and minerals. The health of the human body and other entities depends on temperament’s balance. Any changes in the different states may cause ferocious effects on the human body. The temperament has been determined to play a vital role in determining the person’s normal state (the social, mental, and physical states) and the natural disease process [31].

The process of treatment in the Unani system is orderly. Initially, establishing the therapeutic regimen to normalize the balance of external factors (i.e., food, water, and air) involved in diseases and ailments takes priority. Other means that involve treatment with natural medicines are recommended if the latter does not bear any fruits. The prescriptions by the healer are meant to boost and restore the health and well-being of the patients [32]. The healer also uses various therapeutic approaches. One of them is dietotherapy (Ilaj-bi-ghiza), which involves recommending a specific diet to the patient. It is usually a natural course of treatment in the simplest form. In the illustration, in case of fever, the hakim may recommend a nutrient-rich and low-roughage remedy composed of porridge (Daria) and milk broth (kheer). The therapy takes into consideration the amount and the quality of food. Another intervention that may be used is organotherapy, which entails using organs from healthy animals to heal the same diseased organ in the human body. The tissue of the diseased organ is extracted from the healthy organ in the animal [32].

Similarly, pharmacotherapy (Ilaj-bi-dawa), involving the use of medicines, has identified pharmacotherapy to be natural, less intrusive, more effective, and more environmentally friendly than most methods used. Studies have established that more than 2000 medicines are utilized in this system and extracted from different animal, mineral, and herbal sources [33].

### 2.4. Homeopathy

Homeopathy has been utilized as a healing system in the traditional Indian medical system for over a century [34]. The renowned Dr. Samuel Hahnemann is the protagonist of this system, who popularized it in the mid 17 and 18 centuries. The basic principle of this system is, “like can cure like.” If a substance can elicit symptoms in the human body, it can also cure the same symptoms in sick people. The principle has been around for some time, but it was until 200 years ago that it was applied in medicine. Hahnemann believed that the correct and accurate remedy presented the body’s vital force with mild disease. For instance, if the disease itself is so strong, it overwhelms the ability of the vital force of the body to correct it. The used substances to treat the initial infections are gentle so that it mounts a response that can correct the imbalance existing in the body due to the disease. Since the symptoms produced by the disease are similar to those induced by the medical disease, the body’s responses can lead to healing. The principles of this system are like those of vaccination. A response caused by the disease will protect the body against any amount of disease that can stimulate the body’s response. The difference between vaccination and homeopathy is that the body responds to the vibration of energy instead of a product of disease, preventing both infectious and non-infectious diseases [35].

### 2.5. Yoga

Yoga has become popular in all parts of the world in recent decades. The practice dates to antiquity and has been established as useful to establish physical and spiritual balance in the body. The relevance of yoga in health care has increased as more attention has been paid to the practice. Published reports have indicated that yoga has helped many master the challenges in their lives and to develop confidence and control of their lives. The roots of yoga trace back to ancient India. In describing the meaning of the word yoga, union, mastery, and control are of particular importance. Cultural and individual factors influence the practice of yoga. The practice of yoga requires the subject’s willingness to practice and study while being dedicated and compliant to yoga. Lifestyle changes may be necessary to meet the objectives set [36]. Multiple studies examined the effects of yoga on the physical fitness aspects such as muscular endurance, balance, flexibility, and spinal mobility. Cognitive functions have also been studied, such as attention span, memory, processing speed, and executive function [36].

Different forms of yoga such as Iyengar and Hatha yoga combined with different measures such as the standard sit and reach test have been used to assess the physical flexibility [37]. After several weeks of observation, it was found out that the flexibility of the involved muscles increased over time. Two studies focused on particular postures and the effects they had on specific muscles of the body. One of these studies after three months determined that surface electromyographic signals, while performing the different poses, suggested that the downward facing poses were the most effective to strengthen abdominal muscles [38]. The gluteus maximus muscles could be strengthened by the upward facing poses. Another study that focused on the flexibility of the joints showed that there was increased flexibility and balance in those who performed yoga than those who did not. The practice has been compared to other exercises like stretching and strengthening exercises and calisthenics. Slow movements have been determined to be more effective. At the end of some studies, the mobility of the spine and flexibility of the hamstring had increased. Similarly, yoga also increased the cognitive functioning and the execution speed, and thinking had increased in those who practiced yoga [38].

### 2.6. Naturopathy

Naturopathy is a healing system that utilizes natural remedies to treat diseases and illnesses. It entails different methods like nutritional counseling, herbs, massage therapy, exercising, and acupuncture to solve health problems. The major objective of this therapy is to bring healing to the entire persona. This includes the spirit of the person, mind, and body. Additionally, the ultimate goal is to cure the base issue or the root cause of the illness and not only the presenting symptoms. The expert takes about an hour or two to examine the patient before identifying the treatment remedy. The patient may be questioned on their entire health history, lifestyle patterns, quantum, and any reason that may lead to the acquisition of an illness [39].

In contemporary practice, a naturopathic physician must complete a four-year graduate-level course approved by the council of naturopathic medical education. The entire effort of the physician is to utilize the advantages of traditional medicine and other resources to heal the patient. Additionally, it aims to weed the disease from its basic cause and superficial symptoms. At the end of the treatment, the physician explores the emotional, physical, mental, and mental aspects of one’s health [39,40].

Some of the interventions carried out include changes in the diet patterns of the patient, lifestyle behavior, adoption of hydrotherapy, and some minor surgical procedures. The initial naturopathic appointment tends to be longer, where the physician asks lengthy questions that include the history of the illnesses and the lifestyle habits. He or she can carry out laboratory tests to conclude the diagnosis. The cost of drug therapy and interventions differ from one physician to another.

The massage intervention is used to help the patient to be stress-free. It relieves the client of any tension accumulated in the mind and the body via the massage of the specified areas or even the whole body. Acupuncture may induce a little discomfort on the patient, but it has been shown to help reduce the stress that may have accumulated in some part of the body and that triggers any issues related to health. The patient may also go soaking in the sun or a mud bath for some time. The latter does not involve any discomfort but takes time to realize the results [40]. The traditional allopathic procedures utilized by this system may entail some surgical procedures to cure the disease. However, they may be some side effects [41,42].

The effects of the COVID-19 pandemic on the healthcare system have been immense; there are limited health facilities, resources, and medical therapeutics to address the symptoms [43]. The current interventions are environmental- or pathogen-focused. Optimizing the host’s immunity is important to regulate the effects of the host and reduce the potency of the agent. Apart from the use of vaccines, other ways can also be used to increase the host’s immunity to fight against the viruses. Alternative interventions have made it easier for traditional medicine like Ayurveda and yoga to come into play. There has been immense research into the effects of yoga and Ayurveda on respiratory health, immunity, and mental health. Inarguably, traditional medicine has played a prophylactic and add-on management option for the pandemic [44].

In territories that relied on traditional medicine like Kerala, a state in India successfully flattened the COVID-19 curve in its initial stages. Kerala is the home of Ayurveda medicine, and it heavily implemented its use to fight the malady. The government advised and empowered Ayurveda medicine in the country. The principles of Ayurveda teach the treatment of potent viral and bacterial agents that cause disease. Modern medicine focuses on boosting immunity against potential foreign attacks from agents; the Ayurveda is focused more on the host. It encourages them to adopt a holistic approach to a healthy lifestyle rather than just curing the disease through therapeutic agents [45].

The use of Ayurveda invigorates the users to adopt lifestyle changes and use natural therapies to take control of the balance between the mind, environment, spirit, and body. It usually commences with an internal process of purification, then a special diet, herbal remedies, massage, yoga, and meditation. The healer pays particular attention to the doshas and prescribes herbal medicine and non-pharmacological interventions like meditation and yoga where needed. The Indian Ayurveda, Yoga and Naturopathy, Unani, Siddha, and Homeopathy (AYUSH) ministry advocated using Kadha of natural substances such as ginger, curcumin, honey, and cumin seeds during the day to boost one’s immunity [45]. Based on these preliminary results, the local Kerala government has collaborated with other public health organizations to expand the use of traditional medicine in the treatment of the COVID-19 pandemic. It has helped shorten the recovery time and reduce the complications usually experienced [46].

One popular case published from an Ayurvedic doctor in Tamil Nadu, a state in India, where a 43-year-old male, reported to be the first COVID-19 patient to undergo Ayurvedic treatment, has gained much attention. The patient consulted the doctor through the phone and lost taste and smell, had abdominal pains, and body pain. The patient was administered the treatment on 29 March 2020, for seven days. There was a discernible release from the symptoms by day two. On the fifth day, all of the symptoms previously reported were absent, and the nasopharynx test was negative [46].

Despite millions of people receiving Ayurvedic therapy across the globe for treatment or prophylaxis, not much information has been published about it. Several lessons can be learned from the use of the therapies. One is that patients’ health did not deteriorate during the use of the therapy. On average, the time to recover from the symptoms was about seven days. This was a short time compared to the recovery periods reported by the CDC, which was 3 to 6 weeks. The dosha balance was reported to have been maintained effectively by consuming a regulated diet. The slower digestion time induced by the diet stimulated the body’s digestive powers and helped maintain adequate nourishment. The ayurvedic formulations needed may not be available to most of the patients residing outside India; however, the potency of the available formulations cannot be dismissed [47].

AYUSH has established that the benefits of Yoga and Ayurveda to patients in dire need of pulmonary rehabilitation are many, as the therapy does not only focus on relief of symptoms but also improving the quality of life the function of lungs [48]. Consumption of hot food and water plays a significant role in ensuring that digestion and waste removal are effective. The removal of waste from the body is particularly important because it plays a significant role in one’s susceptibility to viral and bacterial infections. Together with the reliance on medication, other medical conditions that lead to allergy, asthma, fever, and medication issues can be treated. Spices such as ginger, nut-grass rhizomes, coriander, fennel seeds, catechu barks, and cinnamon can be added to boiling water and consumed during the day to provide prophylactic protection to the patients [49].

On close observation, many herbs used in Ayurveda medicine have been shown to interact with ACE2 receptors and spike glycoprotein and have since caused a pharmacologic effect on SARS-CoV-2; they can therefore be relied upon as potent agents to fight the virus. The WHO also confirmed the importance of using alternative medicine to adjuvant therapy in managing COVID-19 [50].

## 3. Traditional Chinese Medicine

**Figure 3:**
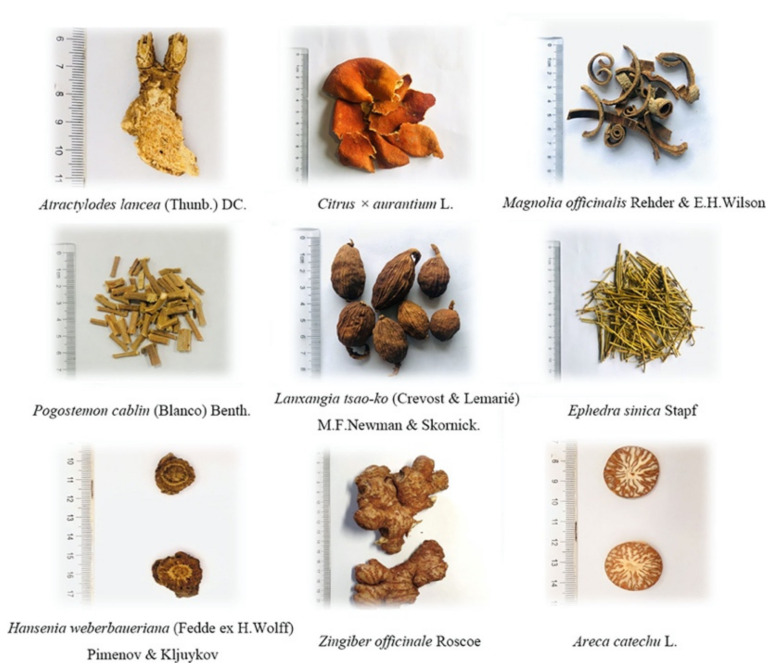
Some of the herbs used in Traditional Chinese Medicine. Reproduced under the terms of the Creative Commons Attribution License: Zhang XR, Li TN, Ren YY, et al. The Important Role of Volatile Components From a Traditional Chinese Medicine Dayuan-Yin Against the COVID-19 Pandemic. *Front Pharmacol* **2020**, 11, 583651. doi:10.3389/fphar.2020.583651.

This system dates back several thousand years. It is grounded in multiple philosophies, including Buddhism, Taoism, and Confucianism and focuses on maintaining and regaining the holistic balance of the body by utilizing natural medicines obtained from minerals, and plants (Figure 3).

Physical maneuvers like acupuncture, massage therapy, among others, are also incorporated in the treatment [51]. One of the most prominent beliefs held by the system is the prevention of disease and secondary infections after the treatment. This system also holds the theory that the body contains four different systems: Wei (defense), qi (vital energy), ying (nutrients), and Xue (blood) [52]. The interaction of these four components can describe a pathology and guide the interventions accordingly. Concentrations of medicines obtained from various plants and herbs, usually boiled, are also used in the treatment process. The system seeks to reinforce qi and is made strong by boosting the defense mechanism and maintaining the body’s homeostasis at equilibrium. This system’s diagnostic tools include some modern techniques integrated with traditional Chinese methods such as listening, physical inspection of the clients, and touching to identify the causes of disease [52].

Two basic ideologies represent the whole process. Homeostasis focuses on maintaining the body’s integrity and the establishment of the closely knitted relationships between the body and the natural and social environment. The second ideology focuses on the dynamic balance that occurs in maintaining integrity. The TCM uses cybernetic ways and discrimination of the systems to recognize the human body. The audio-visual information obtained from the body is analyzed based on the discrimination approach. The human body contains different but complicated systems that interact to maintain integrity. The integrity between the body and the external social and natural environment makes it possible to make inferences from the external information obtained from the body [53].

As an illustration, the heart is the center of the cardiovascular system; other components include the blood, blood vessels, the mind, and the small intestines that collectively form the cardiovascular system and interact with one another to maintain a balance needed in the body. Any information obtained from any part of the system can be used to make conclusions on how the entire system works. In the cybernetic approach, the TCM considers the body as a network of a self-controlled system. The meridians connect the systems, and they are spread throughout the body. The connection of the different parts is affected by the flow of blood and vital energy [53]. The body is divided into different parts in the TCM based on five elements: wood, fire, water, metal, and earth. All these systems have different features that can be understood by analyzing the five elements. The body’s physiology is explained by observing the movement and interchanging among the five elements [53,54].

The TCM’s main focus is on the pathogenicity of the social and natural factors that have a close relationship with humans as they affect integrity. Some of these factors are non-direct and non-specific and cannot be easily discerned as agents like viruses and bacteria. The system focuses on analyzing the symptoms and the signs presented by the patient to identify the disturbances among the self-controlled systems, unlike contemporary medicine that seeks to identify the specific pathogens that cause the diseases. Any disturbances on the parts that make up a system can explain the disturbances of the whole system. It is also important to compare the pathologies that occur at different times to obtain more information. The TCM monitors any connections and changes in different parts of the body [53].

The Zheng theory is a diagnostic tool in the TCM. It is a detailed summary of the cause, location, nature, and the development tendency of a condition at a certain phase during its course. It clarifies the interaction between the body’s reactions and the pathogenic factors that lead to illness. It includes multiple basic features; at the macro level, it is composed of interrelated medical signs and symptoms; this includes the pulse condition and the tongue picture. Secondly, it reflects on the constitutional tendency of the patient, disturbances of the organs of the zangfu, qi, blood, channels, and the collaterals. The patterns of the illness are also likely to change at the different stages of the illness due to a change in the conditions of the body during the disease [55]. Different stages of the illness may have the same patterns due to the similarity of the pathogenesis during the disease’s progress. Apart from using the physical cues, the healer may also ask the patients to state how they feel. The practice utilizes the following four major diagnostic methods; listening and smelling, asking, palpation, and inspection to obtain more information. The practitioner is expected to apply their skills to identify the correct Zheng while also considering subjective information. Recently, scientific studies have been carried out to make the four diagnostic tools more objective [56]. The Zheng theory is utilized in the TCM to treat infections with similar patterns (Zhengs). One phenomenon observed in the system is that one preparation can treat several diseases. If the disease shows different Zhengs, different interventions may be used to treat it. It is very common for one disease to be treated with multiple therapies. The healer identifies the correct Zheng by utilizing the observed symptoms and signs obtained by observation and feeling; they are not completely accurate [57].

An illustration of the Zheng theory, dysmenorrhea (menstrual cramps), can be used to exemplify how a diagnosis is made. Women usually present to the clinic before the onset of their menstrual flow with complaints of severe lower abdominal pain, clots in the menstrual flow, and a disinclination to cold. The woman also states that warmth on the lower abdomen alleviates the pain. The practitioner notes a tight pulse and a purplish tongue. Application of the principles of Zheng to identify the illness deduces that it is an interior Zheng and as an excess of cold Zheng based on the location and presentation of the illness [58]. The exact location of the Zheng needs to be established before carrying forward with treatment. This can only be possible through Zangfu Zheng identification. The condition of the illnesses can be summarized by the nature and the location of the illness. Some of the interventions that can be used to treat the Zheng, in this case, include warming of the uterus to mitigate the cold, relieve pain, and decrease the stasis. Most of the practitioners have adopted at least two methods to identify the accurate Zheng [55,58,59].

The city of Wuhan in China was the epicenter of the COVID-19 pandemic. In fighting the virus, the China National Health Commission identified specific guidelines that can be used to manage the infection that entails the symptomatic treatment, the use of antiviral agents, and the application of the principles of traditional Chinese medicine [60]. Fifteen specific oral and injectable TCM agents were identified to be used against the virus. Studies have shown that these agents have compounds that contain broad antiviral and antibacterial properties, and they are more advantageous in treating pneumonia and other diseases [61].

One of the vital principles of TCM utilized in the treatment is the differentiation of the syndromes of the disease. The drugs also contain active ingredients with multi-target effects that make it hard for drug resistance to develop. Several clinical pathways are involved in the modes of action of the TCMs. These properties have made it possible for the TCMs to be used to manage COVID-19 in China and different parts of the world. The role of TCM in treating COVID-19 cannot be overlooked [62].

The National Health Commission (NHC) guidelines have summarized the clinical symptoms of the patients and the corresponding treatment. The clinical stages of disease identified are divided into the observation and clinical stages of treatment (light, medium, severe, critical, and recovery stages) that manifest differently. Based on the TCM, COVID-19 is classified as a “cold-dampness plague” [63]. The virus affects the lungs, the liver, spleen, and the stomach as it develops and spreads throughout the body. The TCM perspective holds that the virus has an expeditious onset, spreads expressively, presents strong infectivity, and has seasonal symptoms. There are several TCMS recommended in the guidelines [64].

## 4. Miscellaneous Natural Products

### 4.1. Curcumin

Curcumin is one of the essential compounds in the turmeric rhizome (Curcuma longa), a yellow-orange crystalline material. It does not have a lot of different kinds. This product has been used as medicine for the last 4000 years, especially in South Asia [65]. It has been named the “Indian saffron” and the “Golden spice” because of its use as a potent agent in medicinal applications. The compound has been used in Unani, Ayurveda, and Chinese medicine. The discovery of the compound dated back 200 years when it was determined as a pure compound in 1842 [66]. There have been numerous studies showing this as a powerful medicinal agent including antimicrobial properties [67].

The geographical locations of its growth have tremendous effects on the quality of the plant and its nutrition composition [68]. It has been used to add flavor to rice, meat, pasta, and vegetable dishes. It is speculated that it has been used for over 2500 years to treat different diseases and illnesses. It has been used in Ayurveda and traditional Chinese medicine for different conditions. The importance of the plant in treatment is from its orange-yellowed color, which is the most potent component [69]. Studies on the plant have determined that curcumin has antimicrobial, antioxidant, anti-inflammatory, antiangiogenic, antiplatelet, and antimutagenic properties. These properties have preventive and protective abilities against various diseases such as autoimmune, neurological, metabolic, liver, lung, and cardiovascular. The examination of the effects of curcumin on health is therefore necessary [69].

Studies on curcumin’s physical, organic, and inorganic chemistry have established that the substance acts through various mechanisms; this knowledge has been used to develop nutraceuticals and curcumin-based therapeutic agents. The studies showed that curcumin could react with free radicals, form, and break down nonconjugated substances in the body. Curcumin has been established to be effective in treating lifestyle and chronic illnesses through its pharmacological and chemo-preventive abilities. It has also been used in traditional Indian medicine to expulse gas, strengthen the body’s energy, alleviate arthritis, improve digestion, and dissolve gall stones. In modern medicine, it has been used to heal wounds, stimulate the immune system, and decelerate aging [70].

The IUPAC name of curcumin is 1,7-bis(4-hydroxy-3-methoxyphenyl)-1,6-heptadien-3,5-dione and known as diferuloylmethane (C21H20O6). The compound has a melting point of 183 degrees Celsius. Keto in an acidic or neutral medium is the most prominent form of curcumin; in an alkaline medium, it exists as enol. The color of the compound changes in different pH. It is bright yellow in a pH between 2.5 and 7.0 while it exists in red color above a pH of 7. Despite the chemical advantage of the curcumin molecules in structure and form, the principal characteristic is their ability to undergo reduction activities and conjugation. This property makes it have poor availability of the compound in the new formulations of the product. The first step in the chemical reaction of the curcumin is reduction and glucuronidation reactions that yield four molecules of heptadiene-3,5-dione. The curcumin reductase in the liver tissues catalyzes the reduction process. In the second metabolic process, UDP glucuronosyltransferases and sulfotransferase catalyze the conjugation reactions of curcumin through sulfation and glucuronidation [71].

Curcumin has limited bioavailability due to its insufficient absorption, high speed of metabolism, and elimination from the body. These properties make it have limited therapeutics effects in the body. However, new methods have been developed to increase its bioavailability. One of the commonly used interventions is in combination with piperine. The bioavailability of curcumin significantly increases with a combination of piperine; the mechanism is through the decreased glucuronidation of curcumin. The structural analogs of curcumin have been established to increase its bioavailability too [70].

Curcumin has been used in the Jiawei-Xiaoyao, a Chinese traditional treatment system, to treat several diseases such as stress, depression, and dyspepsia for thousands of years now. It has also been established that it is effective in treating neurological disease, cardiovascular disease, and other inflammatory diseases. It is also hypothesized to be effective against the pathogenesis of molecular targets to treat and prevent diseases [72]. The modulation of the molecular targets has a role in the development of the disease. Curcumin has been shown to play vital roles in regulating cytokines, kinases, growth factors, metastatic, receptors, and apoptotic molecules in most phases during most diseases’ development. The curcumin structure’s inclination increases its free radical scavenging activities to low-level hydrogenation and a high level of methoxylation [71]. The structure additionally enables the curcumin to have antioxidant, anticancer, and anti-inflammatory effects.

In the quest to find the cure for the novel Coronaviruses, scientists have considered using natural therapies that contain antiviral pharmacological properties in the prophylaxis against the malady. Curcuma longa (Curcumin), the perennial herbaceous, rhizomatic plant of the ginger family, has been in the limelight. The antiproliferative, anti-inflammatory and medical properties have made it possible to monitor its effects on the COVID-19 pandemic. There are three main curcuminoids in the spice bisdemethoxycurcumin, demethoxycurcumin, and curcuminoids-curcumin. Curcumin (diferuloylmethane) is the principal bioactive element in turmeric. It elicits different pharmacological activities, including anti-inflammatory, antiviral, antioxidant, and antibacterial properties. It has effectively treated enveloped viruses that cause influenza A and other respiratory illnesses [73].

Studies have shown that curcumin has been effective against the COVID-19 variant [74]. The supplementation with curcumin modulates pathogens’ inflammation and oxidative stress on the respiratory system. Studies have shown a direct effect of curcumin on the COVID-19 variant. It has been predicted that curcumin interacts with ACE2 protein and S protein and intervenes by inhibiting the entry of the virus into the lung cells. It has been postulated that curcumin may prevent the entry of the virus into the body and its replication [75].

### 4.2. Elderberry (Sambucus nigra)

This flowering plant belongs to the Adoxaceae family and is used as a supplement to treat flu and cold symptoms. The berries of this plant contain anthocyanin that has been shown to possess powerful antioxidant properties. Its therapeutic use has effectively treated obesity, influenza, upper respiratory infection, metabolic syndrome, gingival recession, and hyperlipidemia. The flowers and the plant’s berries contain antibacterial, antioxidant, glucose-lowering anti-inflammatory, and immune-modulating properties. It can be used alone or combined with other agents to treat various health conditions [76].

The elderberry is an excellent source of free and conjugated amino acids, proteins, fiber fractions, vitamins, antioxidants, and unsaturated fatty acids. Its study shows biological activity elements, primary polyphenols, proanthocyanidins, phenolic acids, and flavonols. The presence of phenols makes the elderberry an ideal medicine and has high antioxidant properties [77].

Many factors affect the composition of the elderberry, such as the degree of ripeness, the variety, and climatic and environmental conditions. The protein nutrient composition is 2.7–2.9% in the fruits, 2.4% in the flowers, and the leaves contain 3.3%. The amino acids occur in the free or conjugated form in the leaves and the flowers. The protein component of the elderberry is complete; among 16 of all the amino acids that belong to the plant cannot be synthesized in the body and must be supplied in the diet, while there are nine amino acids in the leaves and the flowers. The lipids of the plant are usually located in the seeds. The seeds have an oil content of around 22.4%, and in meals produced from the seeds, around 15.9%. The fatty acids are dominantly made up of unsaturated fatty acids; linolenic, linoleic, and oleic acids. The dietary fiber is 7.4% of the total carbohydrate content of 18.4%. Some of the fiber fractions found in the fruit include pectic acid, pectin, calcium pectinate, and pectin. An amount of 6.8–11.5% of the fruits are made up of sugars. Vitamins A, B, and C are found in high amounts. Elderberry is an excellent source of flavonols and phenolic acids. Isorhamnetin, kaempferol, and Quercetin are the most predominant ones. Polyphenolic compounds are found in the leaves, flowers, and fruits. The flavanols are tenfold more in the flowers than the fruits and the leaves [78,79].

Studies established that the processing and storage of the products derived from the elderberry altered the amounts of polyphenols. When fresh fruits are blanched, their composition of polyphenols is decreased, while the anthocyanin content increases; this phenomenon has also been observed in the juices. In the first 30 days, polyphenols decreased by around 40%. In the subsequent days, the amount remained unchanged. Wine from the elderberry showed that anthocyanin content decreased while the flavanols and phenolic acids rose. However, the flavonols and phenolic acids from the plant were relatively stable during the 21-month storage time. These amounts of the content often remain higher in the presence of glucose but lower in ascorbic acid and fructose [80].

The elderberry has been used in folk medicine to treat many ailments and diseases. It has been heavily applied in managing respiratory infections, mainly in colds and cases of flu, but also in dislocations, burns, skin rashes, hemorrhoids, stings, insect bites, and swellings, among others. Traditional medicine recommends the utilization of the elderberry to manage respiratory illnesses such as cold and catarrh, influenza. It is also a laxative, anti-inflammatory, diuretic, and diaphoretic agent. Its components tackle the levels of reactive oxygen species in the body, reactive chlorine species, and reactive nitrogen species through its antioxidant activity [5].

A diet consisting of elderberry fruits is likely to be a potential protective agent against the unwanted effects of oxidative stress on the body and growth. The antioxidant activities are exhibited by the elderberry’s extract, it scavenges hydroxyl radicals and 2,2-diphenyl-1-picrylhydrazyl radical (DPPH•) while inhibiting the lipid peroxidation in the linoleic acid emulsion. Nitric oxide radicals have also been effectively scavenged by the extracts [81,82].

The elderberry has many beneficial effects on increasing the activity of antioxidant enzymes in the plasma, such as glutathione and reducing uric acid levels, reducing oxidative stress, and affecting the blood system. A positive effect of uric acid activity at higher levels has been shown to have antioxidative properties. Uric acid can scavenge ROS and chelate metals such as Fe and Cu. One of the properties that make it important for protection against vascular disease is its ability to penetrate the endothelial cells [83].

There is enough evidence suggesting that treatment of colds and influenza has been effective with elderberry extracts. The extracts have exhibited antiviral and antibacterial activity. At higher concentrations, the elderberry inhibited the activity of influenza A. The compounds are directly bound to the H1N1 Viruses led to preventing their entry into the cells and subsequently prevent an infection [81]. The extracts have been observed to help the host avoid losing any weight in the disease process. It also stimulated the local and systemic immune system responses. Other studies have helped to show the strong inhibitory effects of the extracts on the feline immunodeficiency virus. The extracts have antibacterial activity against Gram-positive bacteria that cause frequent upper respiratory infections. The infusions made from the leaves showed great inhibitory effects on the growth of bacteria (*Bacillus subtilis*, *Bacillus megaterium*, *Escherichia coli*, and *Staphylococcus aureus*) and against yeast. There was also antibacterial against nosocomial pathogens, including methicillin-resistant *S. aureus.* The antimicrobial activity of the flower extracts was more pronounced than that of the fruit extracts [84].

The elderberry extracts stimulated the secretion of proinflammatory cytokines IL-1β, IL-6, IL-8, and TNF-α (tumor necrosis factor) and cytokine IL-10. It increased the production of IFN-β interferon associated with upregulation of TLR-3 (toll-like receptor 3). Elderberry-derived polyphenols caused a 50% decrease in the levels of IL-1β that is responsible for the long-term inflammation in chronic illnesses. The elderberry also can increase the number of lymphocytes in the body [85].

The consumption of the elderberry is considered safe if it is eaten when cooked and consumed moderately. It is dangerous to consume it uncooked because of the potential cyanide toxicity. The intake should be cautiously monitored in diabetic patients to promote glucose and insulin metabolism. Some of the side effects that have been reported upon its use include nausea and vomiting, hypokalemia, dehydration, tachycardia, hypotension, and diarrhea [86].

### 4.3. Glutathione

This is an endogenous peptide that possesses both metabolic and oxidative properties. It has been used to prevent neurotoxicity induced by oxaliplatin and cisplatin [87]. It has also been used to prevent other adverse effects caused by radiation therapy and antineoplastic agents and other disorders like poisoning by heavy metals and other elements, corneal disorders, eczema, and liver disorders [88]. Furthermore, glutathione has been employed treat peripheral vascular disorders and idiopathic pulmonary fibrosis. It is a vital extracellular antioxidant of the lungs, and there is a high concentration of the substance in the epithelial lining fluid of the lungs. It has been shown that a deficiency of glutathione can contribute to the damage of the epithelial lining that is witnessed in different lung disorders. Patients with idiopathic pulmonary fibrosis and cystic fibrosis treated with glutathione had better results over time [89].

Two cytosolic enzymes, glutamylcysteine glutamate and GSH synthetase, catalyze glutathione synthesis from cysteine, glycine, and glutamate. Feedback inhibition, availability of cysteine, and enzyme activity have all been shown to affect glutathione synthesis. The role of substances in the metabolism of nutrients, defense against free radicals, and regulation of cellular events are all vital for the body. Deficiency of the substance leads to oxidative stress that plays a role in aging and the pathogenesis of many other illnesses such as liver disease, parkinsonism, Alzheimer’s disease, and sickle cell anemia, among others [90].

There is a panoply of the roles played by glutathione in the body. First, it helps to get rid of free radicals and other reactive species of oxygen from the body directly and indirectly via enzymatic actions. The GSH is oxidized to GSSG and then reduced to GSH by NADPH-dependent glutathione reductase. Glutathione peroxidase catalyzes GSH reduction of hydrogen peroxide and other peroxides. Secondly, GSH reacts with several electrophiles and physiological metabolites such as prostaglandins, leukotrienes, melanins, and estrogen to form mercapturates. Glutathione-S-transferase initiates these reactions. The substance also conjugates with nitrogen oxide to form an S-nitrosoglutathione adduct cleaved by thioredoxin to release NO and GSH. NO and GSH are vital in the hepatic action of insulin-sensitizing agents that regulate the utilization of amino acids, glucose, and lipids. GSH serves as a substrate for formaldehyde dehydrogenase that converts GSH and formaldehyde to form S-formyl-glutathione. Formaldehyde is a carcinogen, and its removal from the body, is of great physiological importance as it is produced from the metabolism of choline, methanol, sarcosine, xenobiotics, and methionine [91].

It is required to convert prostaglandin H2 to prostaglandin D2 and E2 catalyzed by Endoperoxide isomerase. It is also involved in the glyoxalase system that converts methylglyoxal to D-lactate, a pathway often presents in microorganisms. Due to the vital role of glutathione in the body, adequate concentrations are essential for the proliferation of the cells, including the intestinal epithelial cells and lymphocytes. It is also involved in the process of spermatogenesis in males and the maturation of sperms. Similarly, glutathione is important in activating T-lymphocytes and polymorphonuclear leukocytes and the production of cytokines to mount immune responses against foreign pathogens. Since GSH affects the oxidative, which is an important aspect of the disease process, it can prevent ferocious effects induced by the produced chemicals [92].

Glutathione has been used as adjuvant therapy in the treatment of COVID-19 [93]. There are immunoinflammatory mechanisms that have been implicated in the pathophysiology of COVID-19. Oxidative stress is still a key factor in almost all disease processes; it affects the body’s homeostasis. Glutathione and its precursors such as N-acetylcysteine have been considered for treatment because they influence the binding of the viral proteins to the ACE2 receptor proteins in the cells of the hosts that have been implicated in the COVID-19 infection [94]. The SGH is found in the cytosol of most cells in the body. It comprises three substances: glutamate, cysteine, and glycine. It also plays a role in DNA synthesis, a foreign mechanism that viruses used to replicate and induce an infection. It is the first line of defense against oxidative stress caused by foreign pathogens in the epithelial lining of the lower respiratory tract [95].

The concentrations of GSH in the epithelial fluids are 140 times higher than those found in the serum [94]. Low amounts of endogenous GSH have been implicated to be the cause of the pathogenesis of most illnesses that act through inflammation and oxidative stress, COVID-19 being one of them [96]. COVID-19 is more established in populations with natural or pathological depletion of GSH. The precursor is also important in activating other mechanisms that lodge attacks against the pathophysiological pathways of the vital attacks and the inflammation that follows. It can increase the response of the T cells and decrease the plasma levels of TNF. The precursor also has mucolytic properties that strengthen the protection against pulmonary diseases [97].

### 4.4. Medical Mushrooms

Edible mushrooms are known for their nutritional properties, therapeutic potential, and biological activities. They have emerged as important sources of compounds demonstrating antitumor, antioxidant, and antimicrobial properties [98]. Its bioactive properties enable them with an antioxidant capacity to prevent diseases related to increased oxidative stress and the formation of free radicals. The antimicrobial activities of the compounds and extracts from the mushrooms have been well documented over the years. The use of medical mushrooms has a long tradition in Asian countries. Medicinal compounds from the mushrooms are extracted in multiple ways, through cultivation in the farms or collection from the wild. The fruiting bodies of the mushroom are harvested, or the mycelium is cultivated in the fermenters with solid or liquid substrates. Components in the mushroom can mitigate assaults that make the body vulnerable to cardiovascular illnesses, cancer, and neurodegenerative and metabolic disorders [99].

The mushrooms have been an important therapeutic raw material in folk medicine; for instance, the reishi mushroom (Ganoderma lucidum) was regarded as a panacea in traditional Chinese medicine [100]. The therapeutic effects of the traditionally used species have been corroborated with modern research. The species used for food have good amounts of carbohydrates; their structure has chitin that fills the dietary role. They also have large amounts of proteins that contain essential amino acids and may be alternated with animal products. They have low-fat content making them a good source with low calories, but they have adequate stores of polyunsaturated fatty acids (PUFAs) which present many health benefits to the body. The medicinal and dietary uses of the edible mushrooms are supported by the fact that they contain numerous health-promoting and biologically active compounds. They have secondary metabolites that have a range of benefits, such as antiviral, antibacterial, antioxidative, anticancer, the ability to improve the functioning of the cardiovascular system, and anti-inflammatory properties. The most profound application of medical mushrooms is to prevent inflammation [101,102].

Studies have shown that fungal polysaccharides have a positive effect on human health. A disaccharide contained in the edible mushrooms, trehalose, is composed of reserve material and induces protective properties to the cells against denaturation of the proteins. Experiments show that trehalose inhibits the pro-inflammatory proteins, such as cyclooxygenase-2 and inhibits the degeneration of the inhibitors of nuclear transcription factors. Additionally, trehalose decreases the peroxidation of lipids and arachidonic acid from the phospholipids cell membrane induced by oxygen species. Agaricus bisporus, the most common species of mushroom in the world, has around 1–3% of trehalose when dried. The mushroom hyphae are composed of polysaccharides that enhance immunity and the modulation of the immune system’s defenses, the chitin, glucans, and the chitosans. Apart from the components being sources of fiber to the body, they also protect the intestinal mucous membranes. The normal functioning of the immune system depends on the roles of the probiotic bacteria. The fungal polysaccharides are regarded as prebiotics; they stimulate the growth of the normal bacteria in the body [103].

The glucans and the chitosans have shown antilipemic effects as they decrease the LDL cholesterol levels and the absorption of fat in the gut [104]. They also regulate glycemia; therefore, they prevent the development of obesity, diabetes, and cardiovascular diseases. The β-Glucans, usually contained in the Basidiomycota species, are the biological response modifiers because of their broad-spectrum activities in the immune system [105]. Additionally, they have antioxidant properties, decrease carcinogenic elements’ metabolite levels, and prevent DNA damage. They have also been shown to play a role in producing anti-inflammatory and pro-inflammatory cytokines in the body. They have a high binding affinity to the surface of the immune cell receptors with the pattern recognition receptors in the form of pathogen-associated patterns of molecules. Such receptors include dectins-1, toll-like receptors, and complementary receptor 3. Thus, the immune cells’ maturation and proliferation, stimulation, and activation of the natural killer cells and the macrophages can be activated by β-glucans. Lentinan from the Lentinula species is one of the most typical β-glucan and has been used for a long in the medical realm [105].

The amino acids content in the edible mushrooms is also linked to the anti-inflammatory effects of the medical mushrooms; they have been shown to play a role in the metabolism of prostaglandins [106]. The anti-inflammatory properties of the oyster mushroom have been explained by the presence of isoleucine, tyrosine, phenylalanine, and leucine amino acids. The arginine can inhibit the growth of tumor cells and decrease the risk of developing metastases. Patients who have cancer and who are under arginine supplementation have shown to have a stronger immune system, gained body weight, and have a favorable prognosis than patients who are not under any supplementation. Ergothioneine in mushrooms is an essential amino acid and acts as an antioxidant. It is absorbed by the tissues from the food and is essential for cells prone to oxidative stress like the lens of the eye, semen, skin, and the erythrocytes. The presence of histidine derivatives in A. bisporus has been attributed to relieving oxidative stress. Ergothioneine is chemo and radio-protective and has antimutagenic activities [102,107].

Lectins contained in medical mushrooms can bind selectively with the membrane carbohydrates of different types of cells, playing an important role in the regulation of the immune system. They promote the process of adhesion of cells, and some of them have been shown to activate the lymphocytes, while others have strong anti-proliferative properties. Lectins that have been extracted from the mushrooms have been established as inhibiting the proliferation of tumor cells, especially in breast cancer, without inducing any toxicity. It has also been tested for use in treating psoriasis, eye disorders, especially glaucoma and diabetes [107,108].

The fatty acids of the mushrooms can support human anti-inflammatory processes due to their high composition of unsaturated fatty acids. The PUFAs are precursors of eicosanoids which play a role as signaling molecules necessary for cellular response regulation in the muscles, nerve cells, the immune system, and the blood vessels. They maintain an equilibrium between the anti-inflammatory and inflammatory responses. Therefore, having an adequate supply of fatty acids in the diet helps prevent cardiovascular diseases. α-Linolenic acid (ALA) has been an essential component for normal health and basic nutrition; it is a precursor of long-chained PUFAs [107,109].

It also has potent anti-inflammatory activities. Extracts of chanterelle revealed the presence of fatty acids that showed antagonistic activities towards the receptors activated by peroxisome proliferator-activated receptor γ (PPARγ). The latter plays a vital function in the metabolism of carbohydrates and lipids, in the differentiation of the adipocytes, and in monitoring the inflammation processes [107,108,109]. They inhibit insulin-resistance development; they are efficient against diabetes. They showcase a strong ability to the tumor and inflammation-associated diseases. The fatty acids present in the A. bisporus and other mushrooms can protect the body against hormone-dependent cancers of the breasts. The mechanism of action is by inhibiting aromatase activities, an enzyme involved in the synthesis of estrogen [110].

Among the important secondary metabolites, phenolic compounds are the most useful; they are found in fungal fruits. They contain both anti-inflammatory and antioxidative properties. They do this through multiple mechanisms. They can donate electrons that neutralize the reactive oxygen species and protect the cells against any damage. They can also chelate elements such as iron and copper, which are potential oxygen species generators. They protect the body against free radicals like lipoxygenase, NADH oxygenase, cyclooxygenase, and microsomal monooxygenase. Most of the enzymes are involved in inflammation processes. Some of the phenolic compounds found in the different mushrooms include gallic, cinnamic, caffeic acids, and protocatechuic. Caffeic acids are the most prominent phenolic compound in most mushroom species. The latter exhibited both inflammatory and antioxidative properties [111].

Dried edible mushrooms contain high levels of vitamin C, folic acid, thiamine, niacin, and relatively low amounts of α-tocopherol, riboflavin, and β-carotene. Dietary deficiency of folic acids is a common problem; therefore, these diets can be supplemented with edible mushrooms. The mushroom species are also excellent sources of tocopherols and carotenoids. These compounds have powerful anti-inflammatory and anticancer protection of the body. They also inhibit cellular membranes from peroxidation. The carotenoids are very vital for the normal functioning of the eyes with an added role as powerful antioxidants [102,112].

Several mushroom species contain vitamin D precursor ergosterol, ergocalciferol, and other sterols. Lately, as per studies, vitamin D deficiency has been on the rise. Vitamin deficiency can be attributed to diabetes, hypertension, cancer, and inflammation of the intestines that all are characterized by inflammation. Vitamin D is known for its anti-inflammatory properties. It inhibits the translocation of NF-κB to the nucleus of the cells; this prevents the expression of the pro-inflammatory genes [112].

Due to the well-known antiviral properties of the medical mushroom, they have been explored as potential therapy in fight against COVID-19 [113,114,115].

### 4.5. Astaxanthin

Astaxanthin (AX) pigment belongs to the xanthophylls family, the oxygenated carotenoid derivatives from the plants by lycopene synthesis. It is one of the major components included in the feeds of crustaceans and salmonids. The main role of the pigment is to provide a desirable reddish color to the organisms reared, as most of them do not have access to natural carotenoids [116]. Apart from the induction of pigments, it has antioxidant properties that have been reported to be stronger than those caused by α-tocopherol and β-carotene. Due to these properties, it has been said to protect the organisms from a wide range of diseases such as different types of cancer, cardiovascular-related illnesses, and diseases related to the immune system [117].

The normal aerobic metabolism in the body produces free radicals and reactive oxygen species. AX reacts with the free radicals to generate innocuous compounds by inhibiting the oxidation reactions [118]. However, apart from acting as antioxidants, the carotenoids can also induce oxidative stress on the cells. The AX antioxidative activity is more than ten times stronger than the other carotenoids [119]. It reacts vigorously with other free radicals, depending on the polyene system length and the terminal rings [120].

AX has been proven effective in managing Helicobacter pylori infections that induce gastritis, stomach cancer, and peptic ulcers in humans. The exact mechanism of action against these microorganisms is not known [121]. Due to known regulatory properties of AX for the expression of pro-inflammatory factors as well as antiviral effects, it has been explored as potential adjuvant therapy for COVID-19 disease [122,123,124].

### 4.6. Andrographis paniculata

Andrographis paniculata, commonly referred to as creat or green chiretta plant has been used as a medicinal food for many centuries now. The roots and leaves of the plants have been used for various medicinal purposes in Asia and Europe. It has been used for medicine because of its ‘cold property’ to remove heat and expel toxins from the body. It is being used to stimulate the immune system and manage myocardial ischemia, respiratory tract infections, and pharyngotonsillitis in modern medicine. It also contains anti-inflammatory, anti-microbial and anti-hyperglycemic, anti-sclerosis, antiplatelet, anticancer, choleretic, and anti-hyperglycemic properties. Its leaves and stems contain active phytochemicals such as flavonoids and diterpenoids [125,126].

The major constituent of the plant is andrographolide (a labdane diterpenoid) and has been used as an herbal medicine in Asia for a long time. It has beneficial effects against virus infection, bacteria dysentery, fever, laryngitis, herpes, and rheumatoid arthritis. The compound protects the body against inflammation by binding to the adenosine A2A receptor, inducing the nuclear factor, subsequently inactivating GSK3β, which causes the upregulated expression of heme oxygenase 1. The process regulates the body against oxidative stress from diseases like diabetes, neurodegenerative diseases, and osteoporosis. It can also control inflammation by regulating the expression of proteins (Dai et al., 2019).

The plant’s active compound has been implicated in treating colitis, long-term inflammatory disease of the intestines with limited treatment options. *A. paniculata* mitigates the extension of inflammation of the intestine in colitis induced by transfer of naïve T cells. It does this by affecting the early proliferation and differentiation of the T cells. Additionally, it decreases cytokine expression and splenic cell counts and CD4+ IFN-γ + T cells within 4–7 weeks of undergoing treatment [126,127].

The active compound of *A. paniculata* has been shown to protect the body against infectious agents that destroy the central nervous system. In the process of a CNS disease, the immune responses that protect the body may turn against the host leading to death and morbidity. The plant can prevent the anti-nociceptive activity on hyperalgesia caused by nitroglycerine administration by inhibiting the action of interleukin 6 (IL-6) in the cerebrum and the expression of TNF-α mRNA in the mesencephalon only. Andrographolide can decrease the LPS-induced expression of the cortical C-X-C and C-C subfamily chemokine in vivo [128]. It can also decrease inflammation of the astrocytes and oxidative stress by mediating the out of the cell signal-regulated kinase and Nrf2-p38-MAPK signaling pathways in the primary astrocytes. Regulating the p38-MAPK signaling pathway can protect the MCAO-induced brain injury. It is also involved in suppressing the generation of free radicals, brain infarction, and the blood-brain barrier disruption. It has also shown tremendous benefits in Alzheimer’s diseases; patients prescribed with andrographolide recover spatial memory, learning performance, and synaptic basal transmission. The compound has also shown neuroprotection on the regulation of the synaptic proteins, reduction of the phosphorylated tau proteins, and the maturation of the amyloid-beta aggregate in the aged degus [128].

Recently, peroxisome proliferator-activated receptor-gamma (PPARγ) agonism has been shown as a new strategy to address alcohol use disorder (AUD) and possibly to other addictive substances [129]. The administration of *A. paniculata* extract or andrographolide activates the transcription factor PPARγ [130].

The extractions from A. paniculata have been implicated in the studies concerning the management of the virus. In the studies, the post-infection treatment of the Calus-3 cells significantly prevented the production of infectious virions as per the assays used. In summary, the experimental evidence favored the andrographolide and *A. paniculata* for the advanced users as a monotherapy and combination with other drugs against the COVID-19 virus [131,132].

### 4.7. Propolis

This is a resinous material well known and collected by the bees from the plant exudates and the buds. It is mixed with the wax, the pollen, and the bee enzymes. The bees use it in smoothening the out internal walls, carver the carcasses of the intruders in the hive to prevent them from decomposing, and sealing the holes present in the honeycombs. Due to its antimicrobial and antiseptic properties, it protects the colonies of bees from infection. Since antiquity, propolis has been used by different civilizations to manage colds, ulcers, and wounds because of its local anesthetic and antiseptic properties. It was used to embalm the death by the Egyptians and recently in the Boer War to regenerate the tissues and heal wounds. Its uses have also been applied in contemporary medicine owing to its anti-inflammatory, antioxidant, antitumor, and immune-modulatory activities. Its chemical and biological compositions have been studied to gain more insight into it [133,134].

The use of propolis has not shown any notable side effects after administration. No alterations in enzyme activities were reported. After administration, only a few minor side effects have been reported in treating stomatitis, prevention of otitis media, and mouth ulcers. It protects the renal tissue against free radicals, toxicity, and other adverse effects caused by diatrizoate. Some of the different forms of propolis from Taiwan protected the liver from developing fibrosis [134].

Propolis may directly act on microorganisms in vitro. It can stimulate the immune system in vivo and activate the mechanisms involved in killing microorganisms. The use of antimicrobial drugs with propolis is still being investigated. Findings indicated the ability of propolis to decrease the resistance of the walls of the bacteria to the therapeutically administered antibiotics and caused synergistic effects when administered with antibiotics on the ribosome but do not have any interaction with drugs acting on the DNA or folic acid [135]. It also shows antiviral properties. It exerts the antiviral action by partially blocking the entry of viruses within the cells; this affects the replication steps of the virus into the cells and leads to the degradation of the RNA before the virus enters into the cells or after supernatant is released by the cells. Antifungal properties of propolis have been documented as well; it inhibits the aflatoxigenic fungi, decreasing the percentage of germination of conidia in isolates of flavus. Some of the different types also exerted effects against Candida albicans [135].

Several studies investigating the use of propolis against COVID-19 are reported. Prior studies investigated the in vitro effect of flavonoids on several viruses, including coronavirus. Kaempferol and chrysine were highly potent in preventing the replications [89]. Special attention has been paid to quercetin, a flavanol in propolis; in combination with vitamin C, it has been shown to inhibit aminopeptidase. Quercetin and some of its derivatives inhibit in vitro the proteases involved in COVID-19 pathogenesis. The derivative also modulates the cellular unfolded protein response (UPR). Through the modulation of this pathway, Quercetin may have potential anti-coronavirus effects [136,137].

A potential pharmacological approach to treat COVID-19 aims at downstream effectors like p21-activated kinases. Caffeic acid phenethyl ester (CAPE), one of the most beneficial components of propolis, has shown the ability to downgrade the RAC, a signaling protein in human cells [90]. It is hypothesized to act as a RAC/CDC42-activated inhibitor of the kinase. This experimental evidence suggests that CAPE can help inhibit fibrosis induced by the COVID-19 virus. However, hypersensitivity reactions should be considered when using it for treatment [136,138].

### 4.8. Probiotics

Probiotics are living nonpathogenic microorganisms taken to maintain microbial balance, especially in the gastrointestinal tract. They are composed of Saccharomyces boulardii yeast or lactic acid bacteria such as Bifidobacterium and Lactobacillus species, usually through diet supplements and foods. They exert their beneficial effects via various mechanisms like lowering the pH of the intestines, preventing and decreasing invasion by pathogenic organisms, and modifying the hosts’ immune response [139,140,141].

Not all species of probiotics have all the beneficial effects. The best-documented effectiveness of probiotics is in treating acute diarrhea caused by pouchitis and rotavirus. However, there is a knowledge gap in clarifying the roles of probiotics in preventing diarrhea related to the use of antibiotics, irritable bowel syndrome, Crohn’s disease, vulvovaginal candidiasis, and traveler’s diarrhea. When ingested, there is no consensus on the least number of microorganisms to obtain a beneficial effect. Some scientists are looking into using probiotics to address COVID-19 [142,143]. A recently published report on a randomized, quadruple-blinded, placebo-controlled trial reported a notable increase in specific IgM and IgG against SARS-CoV-2 using a four-strain probiotic composition, indicating its effects on host’s immune system. The COVID-19 outpatients and early viral and symptomatic remission [144].

### 4.9. N-Acetyl Cysteine (NAC)

For decades, N-acetyl cysteine has been used to treat multiple disorders such as intoxication of paracetamol, stable angina pectoris, acute respiratory distress syndrome, doxorubicin-induced cardiotoxicity, HIV/AIDS, radio-contrast induced nephropathy, toxicity from heavy metals, and psychiatric disorders such as bipolar, addiction, and schizophrenia. It is the precursor of amino acid L-cysteine and is available in intravenous, oral, and inhalation forms. Its toxicity is relatively low and presents mild side effects such as nausea, vomiting, tachycardia, pruritus, and rhinorrhea [145].

Studies on the NAC have shown that it interacts with several pathways of metabolism that include regulation of apoptosis and the cell cycle, progression of tumors, expression of genes, immune modulation, and mitochondrial functions. Its half-life is an average of 5.6 h, and it is cleared in the kidneys. This low bioavailability is due to its N-deacetylation in the mucosa of the intestines and first-pass metabolism in the liver. The plasma acts as a pro-oxidizing medium; a redox exchange reaction occurs between cysteine proteins, NAC, and cystine in the plasma to produce NAC–cysteine, NAC–NAC, and cysteine [146]. The latter goes through the epithelial cell membranes and helps synthesize glutathione, an important antioxidant in several physiological processes in the body [147]. Some of these processes include detoxification of the electrophilic xenobiotics, regulations of the immune responses, leukotriene and prostaglandin metabolism, redox-regulated signal transduction modulation, antioxidant defense, signaling of neurotransmitters, and modulation of the proliferation of the cells [148,149].

**Figure 4:**
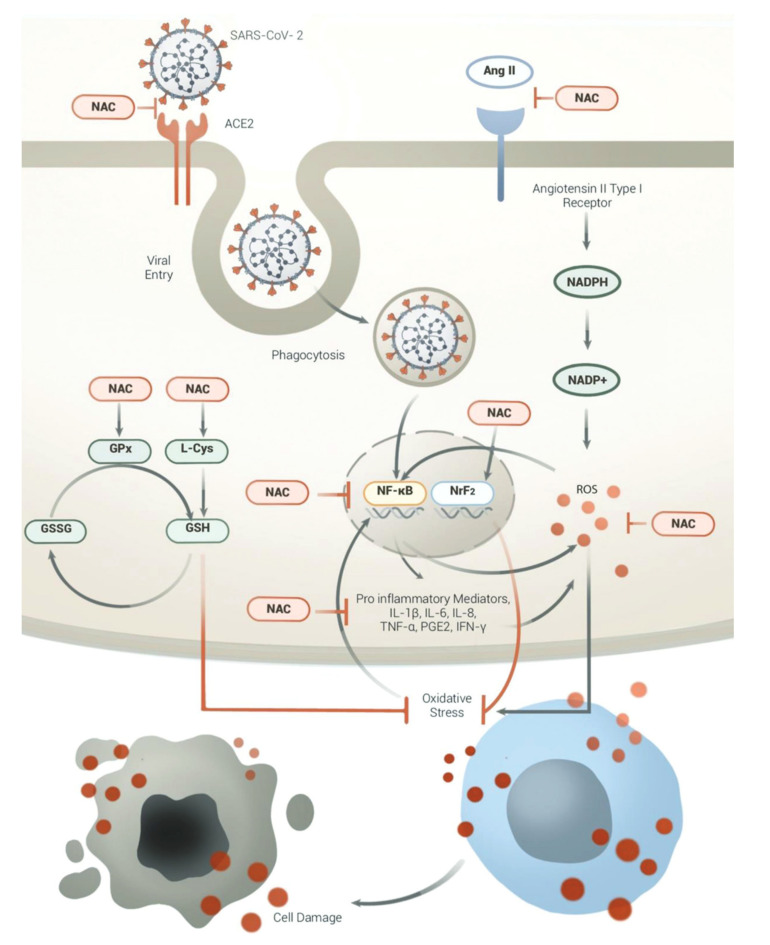
Possible mechanisms of NAC acting as antioxidant and anti-inflammatory agents in SARS-CoV-2 infection. Reproduced under the terms of the Creative Commons Attribution License. Elhidsi, M., Fachrucha, F., & Yudha Irawan, R. (2021). N-Acetylcysteine for COVID-19: A Potential Adjuvant Therapy. Journal of Health Sciences, 11(1), 1–6. https://doi.org/10.17532/jhsci.2020.1156.

N-acetyl cysteine is also known to exhibit antiviral properties [150], not surprisingly, it has also shown great promise in addressing the COVID-19 pandemic mostly by diminishing the cytokine storm and thus preventing acute respiratory distress syndrome (ARDS), the leading cause of death from SARS-CoV-2 infection [151,152,153,154] (Figure 4).

### 4.10. Quercetin

Quercetin is an aglycon flavonoid and is widely found in various fruits, plants, medicinal plants including apples, wild berries, brassica vegetables, tea, as well as in many seeds, nuts, Ginkgo biloba and elderberry, etc. It is an auxin transport inhibitor and reported to exhibit useful pharmacological properties such antioxidant, antiprotozoal, anticancer, antiviral, anti-inflammatory, immunoprotective, antidiabetic, etc. [155,156]. Quercetin exhibits low oral bioavailability (~2%) and gets rapidly eliminated in urine and feces. The absorbed Quercetin goes through II metabolism and gets excreted in urine through kidney and bile through liver [157].

**Figure 5:**
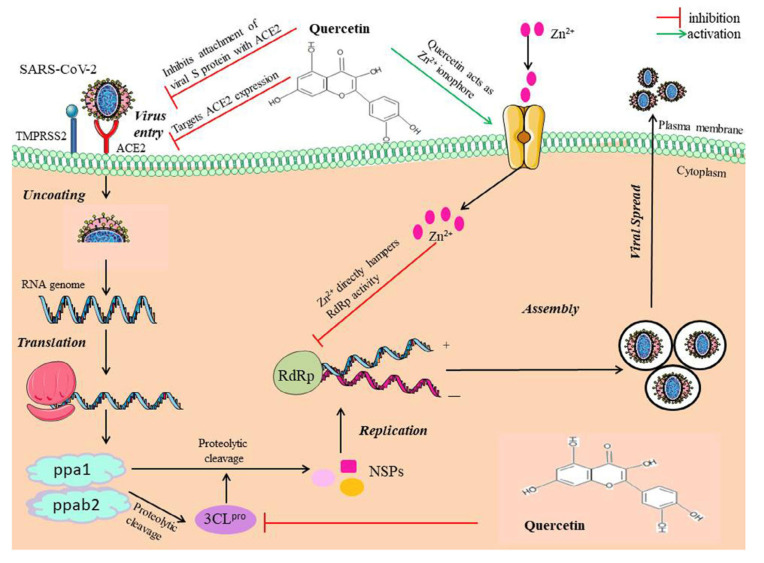
Various mechanisms via which quercetin neutralizes SARS-CoV-2 virus. Reproduced from reference [170] with permission.

Due to its known antiviral properties [158], Quercetin was also extensively evaluated as possible adjuvant therapy and a preventive measure against COVID-19 [159,160,161,162]. Quercetin is a potent nuclear factor erythroid-derived 2-like 2 (NRF2) agonists and these agonists are known to prevent SARS-CoV-2 replication in vitro [163,164]. Quercetin has prevented various zoonotic coronaviruses as well as other viruses’ entry into host cells [165,166]. Quercetin is a potent immune modulator, it inhibits inflammatory pathways leading to cytokine storm, and also reduces the dendritic cells stimulation and expression of major histocompatibility complex (MHC) class II [167]. Quercetin has shown strong inhibitory activity against human angiotensin-converting enzyme 2 receptors (hACE2) expression, thus blocking the SARS-CoV-2 entry in the human cells [168]. Further, querceitin has been shown to target the Chymotrypsin-like Protease (3CL^pro^), Papain-like protease (PL^pro^), and Spike protein (S protein) of the SARS-CoV-2 [169] (Figure 5).

Since Quercetin is a safe and known natural product that has previously demonstrated antiviral capabilities including the novel SARS-CoV-2, it was evaluated in various clinical trials [170]. The results show, using Quercetin alone and in combination with other natural products (curcumin, zinc, vitamin C, etc.) and drugs, results in lessening the clinical manifestations [160,171].

## 5. Conclusions

As the year 2022 is coming to an end, the quest for the most versatile vaccine and antivirals against COVID-19 and its variants still continues. Given the number of new diseases including animal to human transfer that have emerged within the past two decades, it is even more relevant to develop viable, and readily available treatment options to mitigate the progression of these diseases. Natural medicinal compounds in the forms of vegetable and fruits have been consumed for centuries, some cuisines focus a lot more on using spices, condiments, and plant-based oils containing beneficial numerous compounds, leading to a prolonged and healthy life of the consumers. The use of traditional medicine and herbs to optimize the immune system is something that needs to be explored further to bring effective and sustainable therapeutic products into the market quicker. Natural products have a demonstrated history of use in a spectrum of health conditions, owing to a variety of potent immunomodulatory, anti-inflammatory, and antiviral properties. Consuming many of the natural supplements (pure and safe amounts) has shown to decrease susceptibility to viral infections, subsequently improving the clinical outcomes. Medicinal natural products must also be consumed with care and preferably after consultation with a healthcare provider, as some of them are known to cause serious side effects including drug–drug and drug–food interactions. In this article, we have briefly surveyed the traditional medicine systems of India and China, various common nutraceuticals, and natural products, and explored their efficacy in the fight against various diseases and microbial infections, including COVID-19. Although nutraceuticals, like other supportive care options for COVID-19, may not be the sole solution to this pandemic, they may have a profound impact on preventative and supportive measures in these unprecedented times. More clinical trials are warranted to determine the effectiveness of various natural products and capitalize their substantial potential against many diseases and microbial infections, including COVID-19 and the unfortunate emergence of new diseases in the future.

## Data Availability

Not applicable.

## References

[1] Ng T.I., Correia I., Seagal J., DeGoey D.A., Schrimpf M.R., Hardee D.J., Noey E.L., Kati W.M. (2022). Antiviral Drug Discovery for the Treatment of COVID-19 Infections. Viruses.

[2] Nicholson L.B. (2016). The immune system. Essays Biochem..

[3] Soleymani S., Naghizadeh A., Karimi M., Zarei A., Mardi R., Kordafshari G., Esmaealzadeh N., Zargaran A. (2022). COVID-19: General Strategies for Herbal Therapies. J. Evid. Based Integr. Med..

[4] Gombart A.F., Pierre A., Maggini S. (2020). A Review of Micronutrients and the Immune System-Working in Harmony to Reduce the Risk of Infection. Nutrients.

[5] Wieland L.S., Piechotta V., Feinberg T., Ludeman E., Hutton B., Kanji S., Seely D., Garritty C. (2021). Elderberry for prevention and treatment of viral respiratory illnesses: A systematic review. BMC Complement. Med. Ther..

[6] Freckelton I. (2020). COVID-19: Fear, quackery, false representations and the law. Int. J. Law Psychiatry.

[7] Yuan H., Ma Q., Ye L., Piao G. (2016). The Traditional Medicine and Modern Medicine from Natural Products. Molecules.

[8] Calixto J.B. (2019). The role of natural products in modern drug discovery. An. Da Acad. Bras. De Cienc..

[9] Loguercio C., Festi D. (2011). Silybin and the liver: From basic research to clinical practice. World J. Gastroenterol..

[10] Atanasov A.G., Waltenberger B., Pferschy-Wenzig E.M., Linder T., Wawrosch C., Uhrin P., Temml V., Wang L., Schwaiger S., Heiss E.H. (2015). Discovery and resupply of pharmacologically active plant-derived natural products: A review. Biotechnol. Adv..

[11] Negi A.S., Kumar J.K., Luqman S., Shanker K., Gupta M.M., Khanuja S.P. (2008). Recent advances in plant hepatoprotectives: A chemical and biological profile of some important leads. Med. Res. Rev..

[12] Girish C., Pradhan S.C. (2008). Drug development for liver diseases: Focus on picroliv, ellagic acid and curcumin. Fundam. Clin. Pharmacol..

[13] Chandran H., Meena M., Barupal T., Sharma K. (2020). Plant tissue culture as a perpetual source for production of industrially important bioactive compounds. Biotechnol. Rep..

[14] Yeshi K., Crayn D., Ritmejerytė E., Wangchuk P. (2022). Plant Secondary Metabolites Produced in Response to Abiotic Stresses Has Potential Application in Pharmaceutical Product Development. Molecules.

[15] Pye C.R., Bertin M.J., Lokey R.S., Gerwick W.H., Linington R.G. (2017). Retrospective analysis of natural products provides insights for future discovery trends. Proc. Natl. Acad. Sci. USA.

[16] Pandey M.M., Rastogi S., Rawat A.K. (2013). Indian traditional ayurvedic system of medicine and nutritional supplementation. Evid. Based Complement. Altern. Med..

[17] Sen S., Chakraborty R. (2016). Revival, modernization and integration of Indian traditional herbal medicine in clinical practice: Importance, challenges and future. J. Tradit. Complement. Med..

[18] Mishra A., Bentur S.A., Thakral S., Garg R., Duggal B. (2021). The use of integrative therapy based on Yoga and Ayurveda in the treatment of a high-risk case of COVID-19/SARS-CoV-2 with multiple comorbidities: A case report. J. Med. Case Rep..

[19] Kessler C., Wischnewsky M., Michalsen A., Eisenmann C., Melzer J. (2013). Ayurveda: Between religion, spirituality, and medicine. Evid. Based Complement. Altern. Med..

[20] Ragozin B.V. (2016). The history of the development of Ayurvedic medicine in Russia. Anc. Sci. Life.

[21] Warrier M. (2009). Seekership, Spirituality and Self-Discovery: Ayurveda Trainees in Britain. Asian Med..

[22] Pan S.Y., Litscher G., Gao S.H., Zhou S.F., Yu Z.L., Chen H.Q., Zhang S.F., Tang M.K., Sun J.N., Ko K.M. (2014). Historical perspective of traditional indigenous medical practices: The current renaissance and conservation of herbal resources. Evid. Based Complement. Altern. Med..

[23] Patwardhan B., Warude D., Pushpangadan P., Bhatt N. (2005). Ayurveda and traditional Chinese medicine: A comparative overview. Evid. Based Complement. Altern. Med..

[24] Jaiswal Y.S., Williams L.L. (2017). A glimpse of Ayurveda—The forgotten history and principles of Indian traditional medicine. J. Tradit. Complement. Med..

[25] Telles S., Pathak S., Kumar A., Mishra P., Balkrishna A. (2015). Ayurvedic doshas as predictors of sleep quality. Med. Sci. Monit..

[26] Ravishankar B., Shukla V.J. (2007). Indian systems of medicine: A brief profile. Afr. J. Tradit. Complement. Altern. Med..

[27] Hausman G.J. (2022). Dimensions of Authenticity in Siddha Medical and Clinical Research. Asian Med..

[28] Thas J.J. (2008). Siddha medicine—Background and principles and the application for skin diseases. Clin. Dermatol..

[29] Poulakou-Rebelakou E., Karamanou M., George A. (2015). The impact of ancient Greek medicine in India: The birth of Unani medicine. Acta Med. Hist. Adriat..

[30] Sana S.H. (2019). Holistic Approach of Unani Medicine: Integrating Basic Concepts of Unani Medicine and Health Perspective. Int. J. Res. Rev..

[31] Jabin F. (2011). A guiding tool in Unani Tibb for maintenance and preservation of health: A review study. Afr. J. Tradit. Complement. Altern. Med..

[32] Ansari A.P., Ahmed Z.N., Wadud A., Arif M., Khanday S. (2018). Ilaj bil Ghiza (Dietotherapy): A core mode of Unani treatment. J. Adv. Res. Pharm. Sci. Pharmacol. Interv..

[33] Ansari A.P. (2020). ’Ilāj bi’l-Tadbīr (regimenal therapy): A core mode of Unani treatment. J. Complement. Integr. Med..

[34] Ghosh A.K. (2010). A short history of the development of homeopathy in India. Homeopathy.

[35] Loudon I. (2006). A brief history of homeopathy. J. R. Soc. Med..

[36] Field T. (2016). Yoga research review. Complement. Ther. Clin. Pract..

[37] Cramer H., Ward L., Steel A., Lauche R., Dobos G., Zhang Y. (2016). Prevalence, Patterns, and Predictors of Yoga Use: Results of a U.S. Nationally Representative Survey. Am. J. Prev. Med..

[38] Park C.L., Braun T., Siegel T. (2015). Who practices yoga? A systematic review of demographic, health-related, and psychosocial factors associated with yoga practice. J. Behav. Med..

[39] Horrigan B., Lewis S., Abrams D.I., Pechura C. (2012). Integrative Medicine in America—How Integrative Medicine Is Being Practiced in Clinical Centers Across the United States. Glob. Adv. Health Med..

[40] Fleming S.A., Gutknecht N.C. (2010). Naturopathy and the primary care practice. Prim. Care..

[41] Tabish S.A. (2008). Complementary and Alternative Healthcare: Is it Evidence-based?. Int. J. Health Sci..

[42] Galeazzi R.L. (1992). Unerwünschte Wirkungen von Naturheilmitteln [Adverse reactions to natural remedies]. Ther. Umsch..

[43] Kaye A.D., Okeagu C.N., Pham A.D., Silva R.A., Hurley J.J., Arron B.L., Sarfraz N., Lee H.N., Ghali G.E., Gamble J.W. (2021). Economic impact of COVID-19 pandemic on healthcare facilities and systems: International perspectives. Best Pract. Res. Clin. Anaesthesiol..

[44] Umesh C., Ramakrishna K.K., Jasti N., Bhargav H., Varambally S. (2022). Role of Ayurveda and Yoga-Based lifestyle in the COVID-19 pandemic - A narrative review. J. Ayurveda Integr. Med..

[45] Joseph S.M., Iyer D.S., Pillai R.V. (2021). Ayurvedic Response to COVID-19 Pandemic in Kerala, India and Its Impact on Quarantined Individuals—A Community Case Study. Front. Public Health.

[46] Girija P., Sivan N. (2020). Ayurvedic treatment of COVID-19/SARS-CoV-2: A case report. J. Ayurveda Integr. Med..

[47] Maurya V.K., Kumar S., Prasad A.K., Bhatt M., Saxena S.K. (2020). Structure-based drug designing for potential antiviral activity of selected natural products from Ayurveda against SARS-CoV-2 spike glycoprotein and its cellular receptor. Virusdisease.

[48] Tillu G., Salvi S., Patwardhan B. (2020). AYUSH for COVID-19 management. J. Ayurveda Integr. Med..

[49] Yashin A., Yashin Y., Xia X., Nemzer B. (2017). Antioxidant Activity of Spices and Their Impact on Human Health: A Review. Antioxidants.

[50] Khanna K., Kohli S.K., Kaur R., Bhardwaj A., Bhardwaj V., Ohri P., Sharma A., Ahmad A., Bhardwaj R., Ahmad P. (2021). Herbal immune-boosters: Substantial warriors of pandemic COVID-19 battle. Phytomedicine.

[51] Xu J., Xia Z. (2019). Traditional Chinese Medicine (TCM)—Does its contemporary business booming and globalization really reconfirm its medical efficacy & safety?. Med. Drug Discov..

[52] Ma Y., Chen M., Guo Y., Liu J., Chen W., Guan M., Wang Y., Zhao X., Wang X., Li H. (2019). Prevention and treatment of infectious diseases by traditional Chinese medicine: A commentary. Acta Pathol. Microbiol. Immunol. Scand..

[53] Lu A.P., Jia H.W., Xiao C., Lu Q.P. (2004). Theory of traditional Chinese medicine and therapeutic method of diseases. World J. Gastroenterol..

[54] Chung S., Cha S., Lee S.Y., Park J.H., Lee S. (2017). The five elements of the cell. Integr. Med. Res..

[55] Fang J., Zheng N., Wang Y., Cao H., Sun S., Dai J., Li Q., Zhang Y. (2014). Understanding Acupuncture Based on Evidence-Based ZHENG: A Traditional Chinese Medicine Syndrome 2013. Evid Based Complement Altern. Med..

[56] Dai J., Sun S., Cao H., Zheng N., Wang W., Gou X., Su S., Zhang Y. (2012). Applications of New Technologies and New Methods in ZHENG Differentiation. Evid. Based Complement. Alternat. Med..

[57] Xi S., Li Y., Yue L., Gong Y., Qian L., Liang T., Ye Y.A. (2020). Role of Traditional Chinese Medicine in the Management of Viral Pneumonia. Front. Pharmacol..

[58] Wang T., Dong J. (2017). What is “zheng” in traditional Chinese medicine?. J. Tradit. Chin. Med. Sci..

[59] Luiz A.B., Cordovil I., Filho J.B., Ferreira A.S. (2011). *Zangfu zheng* (patterns) are associated with clinical manifestations of *zang shang* (target-organ damage) in arterial hypertension. Chin. Med..

[60] Qiu T., Liang S., Dabbous M., Wang Y., Han R., Toumi M. (2020). Chinese guidelines related to novel coronavirus pneumonia. J. Mark. Access Health Policy.

[61] Ren X., Shao X.X., Li X.X., Jia X.H., Song T., Zhou W.Y., Wang P., Li Y., Wang X.L., Cui Q.H. (2020). Identifying potential treatments of COVID-19 from Traditional Chinese Medicine (TCM) by using a data-driven approach. J. Ethnopharmacol..

[62] Ni L., Chen L., Huang X., Han C., Xu J., Zhang H., Luan X., Zhao Y., Xu J., Yuan W. (2020). Combating COVID-19 with integrated traditional Chinese and Western medicine in China. Acta Pharm. Sin. B.

[63] Zhao F., Yang Z., Wang N., Jin K., Luo Y. (2021). Traditional Chinese Medicine and Western Medicine Share Similar Philosophical Approaches to Fight COVID-19. Aging Dis..

[64] Lyu M., Fan G., Xiao G., Wang T., Xu D., Gao J., Ge S., Li Q., Ma Y., Zhang H. (2021). Traditional Chinese medicine in COVID-19. Acta Pharm. Sin. B.

[65] Sharifi-Rad J., Rayess Y.E., Rizk A.A., Sadaka C., Zgheib R., Zam W., Sestito S., Rapposelli S., Neffe-Skocińska K., Zielińska D. (2020). Turmeric and Its Major Compound Curcumin on Health: Bioactive Effects and Safety Profiles for Food, Pharmaceutical, Biotechnological and Medicinal Applications. Front. Pharmacol..

[66] Kunnumakkara A.B., Bordoloi D., Padmavathi G., Monisha J., Roy N.K., Prasad S., Aggarwal B.B. (2017). Curcumin, the golden nutraceutical: Multitargeting for multiple chronic diseases. Br. J. Pharmacol..

[67] Adamczak A., Ożarowski M., Karpiński T.M. (2020). Curcumin, a Natural Antimicrobial Agent with Strain-Specific Activity. Pharmaceuticals.

[68] Dosoky N.S., Setzer W.N. (2018). Chemical Composition and Biological Activities of Essential Oils of Curcuma Species. Nutrients.

[69] Quispe C., Herrera-Bravo J., Javed Z., Khan K., Raza S., Gulsunoglu-Konuskan Z., Daştan S.D., Sytar O., Martorell M., Sharifi-Rad J. (2022). Therapeutic Applications of Curcumin in Diabetes: A Review and Perspective. BioMed Res. Int..

[70] Patel S.S., Acharya A., Ray R.S., Agrawal R., Raghuwanshi R., Jain P. (2020). Cellular and molecular mechanisms of curcumin in prevention and treatment of disease. Crit. Rev. Food Sci. Nutr..

[71] Nelson K.M., Dahlin J.L., Bisson J., Graham J., Pauli G.F., Walters M.A. (2017). The Essential Medicinal Chemistry of Curcumin. J. Med. Chem..

[72] Hu J., Teng J., Wang W., Yang N., Tian H., Zhang W., Peng X., Zhang J. (2021). Clinical efficacy and safety of traditional Chinese medicine Xiao Yao San in insomnia combined with anxiety. Medicine.

[73] Bormann M., Alt M., Schipper L., van de Sand L., Le-Trilling V.T.K., Rink L., Heinen N., Madel R.J., Otte M., Wuensch K. (2021). Turmeric Root and Its Bioactive Ingredient Curcumin Effectively Neutralize SARS-CoV-2 In Vitro. Viruses.

[74] Pawar K.S., Mastud R.N., Pawar S.K., Pawar S.S., Bhoite R.R., Bhoite R.R., Kulkarni M.V., Deshpande A.R. (2021). Oral Curcumin with Piperine as Adjuvant Therapy for the Treatment of COVID-19: A Randomized Clinical Trial. Front. Pharmacol..

[75] Sharma V.K., Prateeksha, Singh S.P., Singh B.N., Rao C.V., Barik S.K. (2022). Nanocurcumin Potently Inhibits SARS-CoV-2 Spike Protein-Induced Cytokine Storm by Deactivation of MAPK/NF-κB Signaling in Epithelial Cells. ACS Appl. Biomater..

[76] Młynarczyk K., Walkowiak-Tomczak D., Łysiak G.P. (2018). Bioactive properties of Sambucus nigra L. as a functional ingredient for food and pharmaceutical industry. J. Funct. Foods.

[77] Marțiș G.S., Mureșan V., Marc R.M., Mureșan C.C., Pop C.R., Buzgău G., Mureșan A.E., Ungur R.A., Muste S. (2021). The Physicochemical and Antioxidant Properties of *Sambucus nigra* L. and *Sambucus nigra Haschberg* during Growth Phases: From Buds to Ripening. Antioxidants.

[78] Perkins-Veazie P., Thomas A.L., Byers P.L., Finn C.E. (2015). Fruit Composition of Elderberry (*Sambucus* spp.) Genotypes Grown in Oregon and Missouri, USA. Acta Hortic..

[79] Vulić J., Vračar L., Šumić Z. (2008). Chemical characterictics of cultivated elderberry fruit. Acta Period. Technol..

[80] Ferreira S.S., Silva P., Silva A.M., Nunes F.M. (2020). Effect of harvesting year and elderberry cultivar on the chemical composition and potential bioactivity: A three-year study. Food Chem..

[81] Tiralongo E., Wee S.S., Lea R.A. (2016). Elderberry Supplementation Reduces Cold Duration and Symptoms in Air-Travellers: A Randomized, Double-Blind Placebo-Controlled Clinical Trial. Nutrients.

[82] Sidor A., Gramza-Michalowska A. (2015). Advanced research on the antioxidant and health benefit of elderberry (*Sambucus nigra*) in food—A review. J. Funct. Foods.

[83] Hawkins J., Baker C., Cherry L., Dunne E. (2019). Black elderberry (*Sambucus nigra*) supplementation effectively treats upper respiratory symptoms: A meta-analysis of randomized, controlled clinical trials. Complement. Ther. Med..

[84] Fink R.C., Roschek B., Alberte R.S. (2009). HIV type-1 entry inhibitors with a new mode of action. Antivir. Chem. Chemother..

[85] Putra W.E., Rifa’i M. (2019). Immunomodulatory Activities of Sambucus javanica Extracts in DMBA-Exposed BALB/c Mouse. Adv. Pharm. Bull..

[86] Ulbricht C., Basch E., Cheung L., Goldberg H., Hammerness P., Isaac R., Khalsa K.P., Romm A., Rychlik I., Varghese M. (2014). An evidence-based systematic review of elderberry and elderflower (Sambucus nigra) by the Natural Standard Research Collaboration. J. Diet. Suppl..

[87] Avan A., Postma T.J., Ceresa C., Avan A., Cavaletti G., Giovannetti E., Peters G.J. (2015). Platinum-induced neurotoxicity and preventive strategies: Past, present, and future. Oncologist.

[88] Mitchell J.B., Russo A. (1987). The role of glutathione in radiation and drug induced cytotoxicity. Br. J. Cancer.

[89] Grey V., Mohammed S.R., Smountas A.A., Bahlool R., Lands L.C. (2003). Improved glutathione status in young adult patients with cystic fibrosis supplemented with whey protein. J. Cyst. Fibros..

[90] Narayanankutty A., Job J.T., Narayanankutty V. (2019). Glutathione, an Antioxidant Tripeptide: Dual Roles in Carcinogenesis and Chemoprevention. Curr. Protein Pept. Sci..

[91] Lushchak V.I. (2012). Glutathione homeostasis and functions: Potential targets for medical interventions. J. Amino Acids.

[92] Burgess J.R., Yang H., Chang M., Rao M.K., Tu C.P., Reddy C.C. (1987). Enzymatic transformation of PGH2 to PGF2 alpha catalyzed by glutathione S-transferases. Biochem. Biophys. Res. Commun..

[93] Linani A., Benarous K., Bou-Salah L., Yousfi M., Goumri-Said S. (2022). Exploring Structural Mechanism of COVID-19 Treatment with Glutathione as a Potential Peptide Inhibitor to the Main Protease: Molecular Dynamics Simulation and MM/PBSA Free Energy Calculations Study. Int. J. Pept. Res. Ther..

[94] Lana J.F.S.D., Lana A.V.S.D., Rodrigues Q.S., Santos G.S., Navani R., Navani A., da Fonseca L.F., Azzini G.O.M., Setti T., Mosaner T. (2021). Nebulization of glutathione and N-Acetylcysteine as an adjuvant therapy for COVID-19 onset. Adv. Redox Res..

[95] Ghezzi P. (2011). Role of glutathione in immunity and inflammation in the lung. Int. J. Gen. Med..

[96] Polonikov A. (2020). Endogenous Deficiency of Glutathione as the Most Likely Cause of Serious Manifestations and Death in COVID-19 Patients. ACS Infect. Dis..

[97] Silvagno F., Vernone A., Pescarmona G.P. (2020). The Role of Glutathione in Protecting against the Severe Inflammatory Response Triggered by COVID-19. Antioxidants.

[98] Anusiya G., Gowthama Prabu U., Yamini N.V., Sivarajasekar N., Rambabu K., Bharath G., Banat F. (2021). A review of the therapeutic and biological effects of edible and wild mushrooms. Bioengineered.

[99] Valverde M.E., Hernández-Pérez T., Paredes-López O. (2015). Edible mushrooms: Improving human health and promoting quality life. Int. J. Microbiol..

[100] Wang L., Li J.Q., Zhang J., Li Z.M., Liu H.G., Wang Y.Z. (2020). Traditional uses, chemical components and pharmacological activities of the genus *Ganoderma*, P. Karst.: A review. RSC Adv..

[101] Sande D., Oliveira G.P., Moura M.A.F.E., Martins B.A., Lima M.T.N.S., Takahashi J.A. (2019). Edible mushrooms as a ubiquitous source of essential fatty acids. Food Res. Int..

[102] Muszyńska B., Grzywacz-Kisielewska A., Kała K., Gdula-Argasińska J. (2018). Anti-inflammatory properties of edible mushrooms: A review. Food Chem..

[103] Rajewska J., Bałasińska B. (2004). Biologically active compounds of edible mushrooms and their beneficial impact on health. Postep. Hig. I Med. Dosw..

[104] Sima P., Vannucci L., Vetvicka V. (2018). β-glucans and cholesterol (Review). Int. J. Mol. Med..

[105] Vetvicka V., Teplyakova T.V., Shintyapina A.B., Korolenko T.A. (2021). Effects of Medicinal Fungi-Derived β-Glucan on Tumor Progression. J. Fungi.

[106] Ayaz F.A., Chuang L.T., Torun H., Colak A., Sesli E., Presley J., Smith B.R., Glew R.H. (2011). Fatty acid and amino acid compositions of selected wild-edible mushrooms consumed in Turkey. Int. J. Food Sci. Nutr..

[107] Sharma D., Singh V.P., Singh N.K. (2018). A Review on Phytochemistry and Pharmacology of Medicinal as well as Poisonous Mushrooms. Mini Rev. Med. Chem..

[108] Coelho L.C.B.B., Silva P.M.D.S., Lima V.L.D.M., Pontual E.V., Paiva P.M.G., Napoleao T.H., Correia M.T.D.S. (2017). Lectins, Interconnecting Proteins with Biotechnological/Pharmacological and Therapeutic Applications. Evid. Based Complement. Alternat. Med..

[109] Fontes A., Alemany-Pagès M., Oliveira P.J., Ramalho-Santos J., Zischka H., Azul A.M. (2019). Antioxidant Versus Pro-Apoptotic Effects of Mushroom-Enriched Diets on Mitochondria in Liver Disease. Int. J. Mol. Sci..

[110] Wińska K., Mączka W., Gabryelska K., Grabarczyk M. (2019). Mushrooms of the Genus Ganoderma Used to Treat Diabetes and Insulin Resistance. Molecules.

[111] Ganeshpurkar A., Rai G., Jain A.P. (2010). Medicinal mushrooms: Towards a new horizon. Pharmacogn. Rev..

[112] Chaturvedi V.K., Agarwal S., Gupta K.K., Ramteke P.W., Singh M.P. (2018). Medicinal mushroom: Boon for therapeutic applications. Biotech.

[113] Hetland G., Johnson E., Bernardshaw S.V., Grinde B. (2021). Can medicinal mushrooms have prophylactic or therapeutic effect against COVID-19 and its pneumonic superinfection and complicating inflammation?. Scand. J. Immunol..

[114] Arunachalam K., Sasidharan S.P., Yang X. (2022). A concise review of mushrooms antiviral and immunomodulatory properties that may combat against COVID-19. Food Chem. Adv..

[115] Slomski A. (2021). Trials Test Mushrooms and Herbs as Anti-COVID-19 Agents. JAMA.

[116] Donoso A., González-Durán J., Muñoz A.A., González P.A., Agurto-Muñoz C. (2021). Therapeutic uses of natural astaxanthin: An evidence-based review focused on human clinical trials. Pharmacol. Res..

[117] Higuera-Ciapara I., Félix-Valenzuela L., Goycoolea F.M. (2006). Astaxanthin: A review of its chemistry and applications. Crit. Rev. Food Sci. Nutr..

[118] Brotosudarmo T.H.P., Limantara L., Setiyono E., Heriyanto (2020). Structures of Astaxanthin and Their Consequences for Therapeutic Application. Int. J. Food Sci..

[119] Ambati R.R., Phang S.M., Ravi S., Aswathanarayana R.G. (2014). Astaxanthin: Sources, extraction, stability, biological activities and its commercial applications—A review. Mar. Drugs.

[120] Fakhri S., Abbaszadeh F., Dargahi L., Jorjani M. (2018). Astaxanthin: A mechanistic review on its biological activities and health benefits. Pharmacol. Res..

[121] Kang H., Kim H. (2017). Astaxanthin and β-carotene in Helicobacter pylori-induced Gastric Inflammation: A Mini-review on Action Mechanisms. J. Cancer Prev..

[122] Talukdar J., Bhadra B., Dattaroy T., Nagle V., Dasgupta S. (2020). Potential of natural astaxanthin in alleviating the risk of cytokine storm in COVID-19. Biomed. Pharmacother..

[123] Ahmadi A.R., Ayazi-Nasrabadi R. (2021). Astaxanthin protective barrier and its ability to improve the health in patients with COVID-19. Iran. J. Microbiol..

[124] Sivarajan R., Oberwinkler H., Roll V., König E.M., Steinke M., Bodem J. (2022). A defined anthocyanin mixture sourced from bilberry and black currant inhibits Measles virus and various herpesviruses. BMC Complement. Med. Ther..

[125] Akbar S. (2011). Andrographis paniculata: A review of pharmacological activities and clinical effects. Altern. Med. Rev..

[126] Dai Y., Chen S.R., Chai L., Zhao J., Wang Y., Wang Y. (2019). Overview of pharmacological activities of *Andrographis paniculata* and its major compound andrographolide. Crit. Rev. Food Sci. Nutr..

[127] Naomi R., Bahari H., Ong Z.Y., Keong Y.Y., Embong H., Rajandram R., Teoh S.H., Othman F., Hasham R., Yin K.B. (2022). Mechanisms of Natural Extracts of *Andrographis paniculata* That Target Lipid-Dependent Cancer Pathways: A View from the Signaling Pathway. Int. J. Mol. Sci..

[128] Lu J., Ma Y., Wu J., Huang H., Wang X., Chen Z., Chen J., He H., Huang C. (2019). A review for the neuroprotective effects of andrographolide in the central nervous system. Biomed. Pharmacother..

[129] Islam M.T. (2017). Andrographolide, a New Hope in the Prevention and Treatment of Metabolic Syndrome. Front. Pharmacol..

[130] Stopponi S., Fotio Y., Cifani C., Li H., Haass-Koffler C.L., Cannella N., Demopulos G., Gaitanaris G., Ciccocioppo R. (2021). Andrographis paniculata and Its Main Bioactive Ingredient Andrographolide Decrease Alcohol Drinking and Seeking in Rats Through Activation of Nuclear PPARγ Pathway. Alcohol Alcohol..

[131] Sa-ngiamsuntorn K., Suksatu A., Pewkliang Y., Thongsri P., Kanjanasirirat P., Manopwisedjaroen S., Charoensutthivarakul S., Wongtrakoongate P., Pitiporn S., Chaopreecha J. (2021). Anti-SARS-CoV-2 Activity of Andrographis paniculata Extract and Its Major Component Andrographolide in Human Lung Epithelial Cells and Cytotoxicity Evaluation in Major Organ Cell Representatives. J. Nat. Prod..

[132] Intharuksa A., Arunotayanun W., Yooin W., Sirisa-Ard P. (2022). A Comprehensive Review of Andrographis paniculata (Burm. f.) Nees and Its Constituents as Potential Lead Compounds for COVID-19 Drug Discovery. Molecules.

[133] Kuropatnicki A.K., Szliszka E., Krol W. (2013). Historical aspects of propolis research in modern times. Evid. Based Complement. Alternat. Med..

[134] da Silva Barboza A., Aitken-Saavedra J.P., Ferreira M.L., Fábio Aranha A.M., Lund R.G. (2021). Are propolis extracts potential pharmacological agents in human oral health?—A scoping review and technology prospecting. J. Ethnopharmacol..

[135] Almuhayawi M.S. (2020). Propolis as a novel antibacterial agent. Saudi J. Biol. Sci..

[136] Berretta A.A., Silveira M., Cóndor Capcha J.M., De Jong D. (2020). Propolis and its potential against SARS-CoV-2 infection mechanisms and COVID-19 disease: Running title: Propolis against SARS-CoV-2 infection and COVID-19. Biomed. Pharmacother..

[137] Dilokthornsakul W., Kosiyaporn R., Wuttipongwaragon R., Dilokthornsakul P. (2022). Potential effects of propolis and honey in COVID-19 prevention and treatment: A systematic review of in silico and clinical studies. J. Integr. Med..

[138] Maruta H., He H. (2020). PAK1-blockers: Potential Therapeutics against COVID-19. Med. Drug Discov..

[139] Williams N.T. (2010). Probiotics. Am. J. Health Syst. Pharm..

[140] Shi L.H., Balakrishnan K., Thiagarajah K., Mohd Ismail N.I., Yin O.S. (2016). Beneficial Properties of Probiotics. Trop. Life Sci. Res..

[141] Plaza-Diaz J., Ruiz-Ojeda F.J., Gil-Campos M., Gil A. (2020). Mechanisms of Action of Probiotics. Adv. Nutr..

[142] Antunes A.E.C., Vinderola G., Xavier-Santos D., Sivieri K. (2020). Potential contribution of beneficial microbes to face the COVID-19 pandemic. Food Res. Int..

[143] Bottari B., Castellone V., Neviani E. (2021). Probiotics and COVID-19. Int. J. Food Sci. Nutr..

[144] Gutiérrez-Castrellón P., Gandara-Martí T., Abreu Y., Abreu A.T., Nieto-Rufino C.D., López-Orduña E., Jiménez-Escobar I., Jiménez-Gutiérrez C., López-Velazquez G., Espadaler-Mazo J. (2022). Probiotic improves symptomatic and viral clearance in Covid19 outpatients: A randomized, quadruple-blinded, placebo-controlled trial. Gut Microbes.

[145] Tenório M.C.D.S., Graciliano N.G., Moura F.A., Oliveira A.C.M., Goulart M.O.F. (2021). N-Acetylcysteine (NAC): Impacts on Human Health. Antioxidants.

[146] Papi A., Di Stefano A.F.D., Radicioni M. (2021). Pharmacokinetics and Safety of Single and Multiple Doses of Oral N-Acetylcysteine in Healthy Chinese and Caucasian Volunteers: An Open-Label, Phase I Clinical Study. Adv. Ther..

[147] Lu S.C. (2013). Glutathione synthesis. Biochim. Biophys. Acta.

[148] Dodd S., Dean O., Copolov D.L., Malhi G.S., Berk M. (2008). N-acetylcysteine for antioxidant therapy: Pharmacology and clinical utility. Expert Opin. Biol. Ther..

[149] Mokhtari V., Afsharian P., Shahhoseini M., Kalantar S.M., Moini A. (2017). A Review on Various Uses of N-Acetyl Cysteine. Cell J..

[150] Schwalfenberg G.K. (2021). N-Acetylcysteine: A Review of Clinical Usefulness (an Old Drug with New Tricks). J. Nutr. Metab..

[151] Wong K.K., Lee S., Kua K.P. (2021). N-Acetylcysteine as Adjuvant Therapy for COVID-19—A Perspective on the Current State of the Evidence. J. Inflamm. Res..

[152] Mohanty R.R., Padhy B.M., Das S., Meher B.R. (2021). Therapeutic potential of N-acetyl cysteine (NAC) in preventing cytokine storm in COVID-19: Review of current evidence. Eur. Rev. Med. Pharmacol. Sci..

[153] Izquierdo J.L., Soriano J.B., González Y., Lumbreras S., Ancochea J., Echeverry C., Rodríguez J.M. (2022). Use of N-Acetylcysteine at high doses as an oral treatment for patients hospitalized with COVID-19. Sci. Prog..

[154] Sanchez-Gonzalez M.A., Moskowitz D., Issuree P.D., Yatzkan G., Rizvi S.A.A., Day K. (2020). A Pathophysiological Perspective on COVID-19’s Lethal Complication: From Viremia to Hypersensitivity Pneumonitis-like Immune Dysregulation. Infect. Chemother..

[155] Yang D., Wang T., Long M., Li P. (2020). Quercetin: Its Main Pharmacological Activity and Potential Application in Clinical Medicine. Oxid. Med. Cell Longev..

[156] Batiha G.E., Beshbishy A.M., Ikram M., Mulla Z.S., El-Hack M.E.A., Taha A.E., Algammal A.M., Elewa Y.H.A. (2020). The Pharmacological Activity, Biochemical Properties, and Pharmacokinetics of the Major Natural Polyphenolic Flavonoid: Quercetin. Foods.

[157] Muñoz-Reyes D., Morales A.I., Prieto M. (2021). Transit and Metabolic Pathways of Quercetin in Tubular Cells: Involvement of Its Antioxidant Properties in the Kidney. Antioxidants.

[158] Di Petrillo A., Orrù G., Fais A., Fantini M.C. (2022). Quercetin and its derivates as antiviral potentials: A comprehensive review. Phytother. Res..

[159] Manjunath S.H., Thimmulappa R.K. (2022). Antiviral, immunomodulatory, and anticoagulant effects of quercetin and its derivatives: Potential role in prevention and management of COVID-19. J. Pharm. Anal..

[160] Imran M., Thabet H.K., Alaqel S.I., Alzahrani A.R., Abida A., Alshammari M.K., Kamal M., Diwan A., Asdaq S.M.B., Alshehri S. (2022). The Therapeutic and Prophylactic Potential of Quercetin against COVID-19: An Outlook on the Clinical Studies, Inventive Compositions, and Patent Literature. Antioxidants.

[161] Munafò F., Donati E., Brindani N., Ottonello G., Armirotti A., De Vivo M. (2022). Quercetin and luteolin are single-digit micromolar inhibitors of the SARS-CoV-2 RNA-dependent RNA polymerase. Sci. Rep..

[162] Aucoin M., Cooley K., Saunders P.R., Cardozo V., Remy D., Cramer H., Abad C.N., Hannan N. (2020). The effect of quercetin on the prevention or treatment of COVID-19 and other respiratory tract infections in humans: A rapid review. Adv. Integr. Med..

[163] Bahoosh S.R., Shokoohinia Y., Eftekhari M. (2022). Glucosinolates and their hydrolysis products as potential nutraceuticals to combat cytokine storm in SARS-COV-2. Daru.

[164] Jia H., Zhang Y., Si X., Jin Y., Jiang D., Dai Z., Wu Z. (2021). Quercetin Alleviates Oxidative Damage by Activating Nuclear Factor Erythroid 2-Related Factor 2 Signaling in Porcine Enterocytes. Nutrients.

[165] Yi L., Li Z., Yuan K., Qu X., Chen J., Wang G., Zhang H., Luo H., Zhu L., Jiang P. (2004). Small molecules blocking the entry of severe acute respiratory syndrome coronavirus into host cells. J. Virol..

[166] Catalano A., Iacopetta D., Ceramella J., Maio A.C., Basile G., Giuzio F., Bonomo M.G., Aquaro S., Walsh T.J., Sinicropi M.S. (2022). Are Nutraceuticals Effective in COVID-19 and Post-COVID Prevention and Treatment?. Foods.

[167] Verna G., Liso M., Cavalcanti E., Bianco G., Di Sarno V., Santino A., Campiglia P., Chieppa M. (2021). Quercetin Administration Suppresses the Cytokine Storm in Myeloid and Plasmacytoid Dendritic Cells. Int. J. Mol. Sci..

[168] Alshrari A.S., Hudu S.A., Imran M., Asdaq S.M.B., Ali A.M., Rabbani S.I. (2022). Innovations and development of COVID-19 vaccines: A patent review. J. Infect. Public Health.

[169] Mouffouk C., Mouffouk S., Mouffouk S., Hambaba L., Haba H. (2021). Flavonols as potential antiviral drugs targeting SARS-CoV-2 proteases (3CL^pro^ and PL^pro^), spike protein, RNA-dependent RNA polymerase (RdRp) and angiotensin-converting enzyme II receptor (ACE2). Eur. J. Pharmacol..

[170] Pawar A., Russo M., Rani I., Goswami K., Russo G.L., Pal A. (2022). A critical evaluation of risk to reward ratio of quercetin supplementation for COVID-19 and associated comorbid conditions. Phytother. Res..

[171] Khan A., Iqtadar S., Mumtaz S.U., Heinrich M., Pascual-Figal D.A., Livingstone S., Abaidullah S. (2022). Oral Co-Supplementation of Curcumin, Quercetin, and Vitamin D3 as an Adjuvant Therapy for Mild to Moderate Symptoms of COVID-19-Results From a Pilot Open-Label, Randomized Controlled Trial. Front Pharmacol.

